# Water‐soluble Self‐assembled {Pd_84_}^Ac^ Polyoxopalladate Nano‐wheel as a Supramolecular Host

**DOI:** 10.1002/anie.202214203

**Published:** 2022-11-30

**Authors:** Zoë L. Sinclair, Nicola L. Bell, Jessica R. Bame, De‐Liang Long, Leroy Cronin

**Affiliations:** ^1^ Digital Chemistry Advanced Research Centre (ARC) University of Glasgow Glasgow G11 6EW UK

**Keywords:** Nuclear Magnetic Resonance, Polyoxopalladate, Supramolecular Chemistry

## Abstract

Polyoxopalladates (POPs) are a class of self‐assembling palladium‐oxide clusters that span a variety of sizes, shapes and compositions. The largest of this family, {Pd_84_}^Ac^, is constructed from 14 building units of {Pd_6_} and lined on the inner and outer torus by 28 acetate ligands. Due to its high water solubility, large hydrophobic cavity and distinct ^1^H NMR fingerprint {Pd_84_}^Ac^ is an ideal molecule for exploring supramolecular behaviour with small organic molecules in aqueous media. Molecular visualisation studies highlighted potential binding sites between {Pd_84_}^Ac^ and these species. Nuclear Magnetic Resonance (NMR) techniques, including ^1^H NMR, ^1^H Diffusion Ordered Spectroscopy (DOSY) and Nuclear Overhauser Spectroscopy (NOESY), were employed to study the supramolecular chemistry of this system. Here, we provide conclusive evidence that {Pd_84_}^Ac^ forms a 1 : 7 host‐guest complex with benzyl viologen (BV^2+^) in aqueous solution.

## Graphical abstract

The largest anionic polyoxopalladate ({Pd_84_}^Ac^) is shown to act as a water‐soluble inorganic host encapsulating organic guests in a Russian Doll fashion. NMR analysis including ^1^H NMR, Diffusion Ordered Spectroscopy (DOSY) and Nuclear Overhauser Spectroscopy (NOESY), as well as ESI‐MS, was used to study the supramolecular chemistry of this system providing conclusive evidence that {Pd_84_}^Ac^ forms a 1 : 7 host‐guest complex with benzyl viologen (BV^2+^) in aqueous solution.

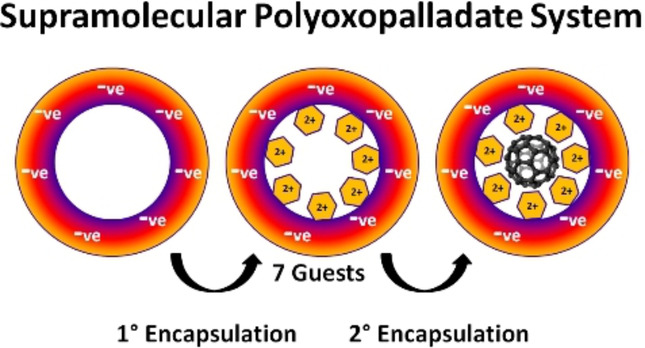

## Introduction

**Figure 1:**
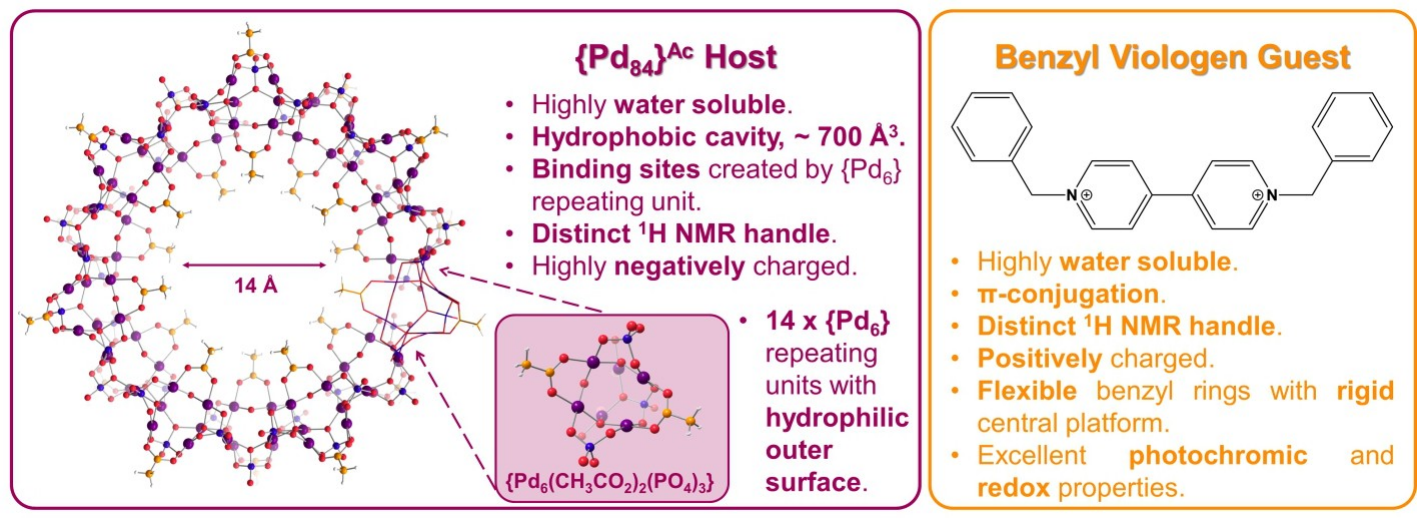
The largest POP to date, the nano‐wheel; {Pd_84_}^Ac^ and benzyl viologen with their characteristics that make them attractive targets for investigation in supramolecular chemistry. Colour scheme: purple, Pd; blue, P; orange, C; and red, O.

Polyoxometalates (POMs) are a class of large, self‐assembling inorganic structures formed by the condensation of metal oxides, usually under acidic conditions.[^1^, ^2^, ^3^] First row transition metals are most commonly used, but later transition metals including Pd and Pt have been incorporated.^[4]^ The most recent advances in polyoxopalladate chemistry reveal neutral and cationic cluster families which differ from the majority of polyoxopalladates which are anionic.[^5^, ^6^, ^7^] In 2012, the largest polyoxopalladate (POP), {Pd_84_}^Ac^ was reported by Cronin et al. With its hydrophobic cavity, multiple binding sites, high water solubility and charge of −70 this nano‐wheel is an excellent candidate for studying host‐guest chemistry.[^8^, ^9^, ^10^] Applications of host‐guest systems include sensing, drug delivery vehicles, molecular machinery and catalysis.[^11^, ^12^, ^13^, ^14^, ^15^, ^16^] Macrocyclic hosts most commonly studied include crown ethers, metal‐organic cages, cyclodextrins, cucurbiturils, calixarenes and resorcinarenes.[^17^, ^18^, ^19^, ^20^, ^21^] The presence of transition metals in these structures allows a greater degree of control over molecular shape, binding sites and chemical selectivity due to the preferred coordination geometry the metal adopts on complexation with a specific ligand.[^22^, ^23^] Metallacycles, cages and MOFs have all attracted attention, whilst the use of polyoxometalates is relatively unexplored.[^24^, ^25^, ^26^, ^27^] Paraquat derivatives (*N*,*N′*‐dialkyl‐4,4′‐bipyridinium salts) such as benzyl viologen (BV^2+^) are well‐known for their strong redox, electrochromic and photochromic properties.[^28^, ^29^, ^30^, ^31^, ^32^] They have extended π‐conjugation and are cationic in nature, making them suitable candidates for binding with anionic host species. We sought to exploit the hydrophobic cavity of the largest palladium‐based POM macrocycle, {Pd_84_}^Ac^, by investigating its interaction with benzyl viologen, BV^2+^ (Figure 1), and characterising it via multiple NMR techniques. Through this investigation and the need for high quality host material we also improved the reliability and robustness of the synthetic procedure for {Pd_84_}^Ac^ which can be found in the Supporting Information.

## Results and Discussion

{Pd_84_}^Ac^ has D_7d_ symmetry and is composed of 14 {Pd_6_} repeating units: {Pd_6_(μ_4_‐O)_2_(μ_2_‐O)(CH_3_CO_2_)_2_(PO_4_)_3_}. In each {Pd_6_} building block six palladium atoms are linked by μ_4_‐O oxo ligands, capped by two phosphate ligands which are on the outer surface of the sub‐unit and bridged by a single phosphate group. One acetate ligand points into the centre of the cavity and one sits on the outer surface. The outer ligands point perpendicular to each other and lie on alternating faces of the {Pd_6_} sub‐units minimising steric hindrance. The arrangement of these ligands on the internal surface of the nano‐wheel creates pockets with an electro‐positive nature. The ^1^H NMR displays a single set of well‐resolved peaks for the carboxylate ligands on the inner and outer rim of {Pd_84_}^Ac^. The inner −CH_3_ protons, **I**, are represented by a peak at 2.53 ppm whilst the outer −CH_3_ protons, **II**, resonate upfield with a broad peak at 1.86 ppm, Figure S5. Hydrolysis occurs on a slow time‐scale relative to the NMR measurement, therefore an equilibrium exists between charge separated (i.e. free) acetate ligands and those bound to palladium centres and there is correspondingly three distinct resonances in the spectrum for the bound ligands (**I** and **II**) and the free ligands.^[33]^ The difference in chemical shift for **I** and **II** arises from the subtle variance in their respective chemical environments. The internal carboxylate ligand methyl tails are near each other compared to those on the outer face. There is a single μ_2_‐O connecting two Pd atoms on the inner surface on the nano‐wheel and two phosphate groups capping the sub‐unit on either side. The outer surface of the nano‐wheel has only a single phosphate which bridges each {Pd_6_}. These dissimilarities make the internal surface slightly more electro‐positive than the external surface corresponding to the slight downfield shift of the inner −CH_3_ due to the de‐shielding effect caused by this electronic environment.

Supramolecular systems rely on a variety of non‐covalent bonds to stabilise the interaction: electrostatic interactions, van der Waals forces, π‐effects and the hydrophobic effect.^[34]^ The polyoxopalladate host, {Pd_84_}^Ac^, with its acetate ligands on the inner and outer surface is a richly hydrophobic environment that is filled with water molecules when it exists alone in solution. Enthalpic hydrophobic interactions occur in systems where there is a single organic (often apolar) guest molecule in aqueous solution within a cavity.[^35^, ^36^, ^37^, ^38^] The guest molecule readily replaces the water in the cavity because the interaction between the cavity of the host and the water molecules is weak, so the energy of the system is very high. This means it is enthalpically favourable for the water to be expelled by the guest molecules, lowering the energy, and stabilising the system. Therefore the addition of an organic molecule would result in an enthalpic hydrophobic effect creating a more stable environment in aqueous solution. Since the binding sites within the {Pd_84_}^Ac^ molecule are in close proximity to one another we predict that the binding event of a single guest would enhance the possibility of further binding through π‐π‐stacking between guest species. This process is known as positive cooperativity and a number of such supramolecular host‐guest systems have been reported using NMR, UV/Vis and calorimetry.[^39^, ^40^, ^41^, ^42^, ^43^, ^44^, ^45^, ^46^]

**Figure 2:**
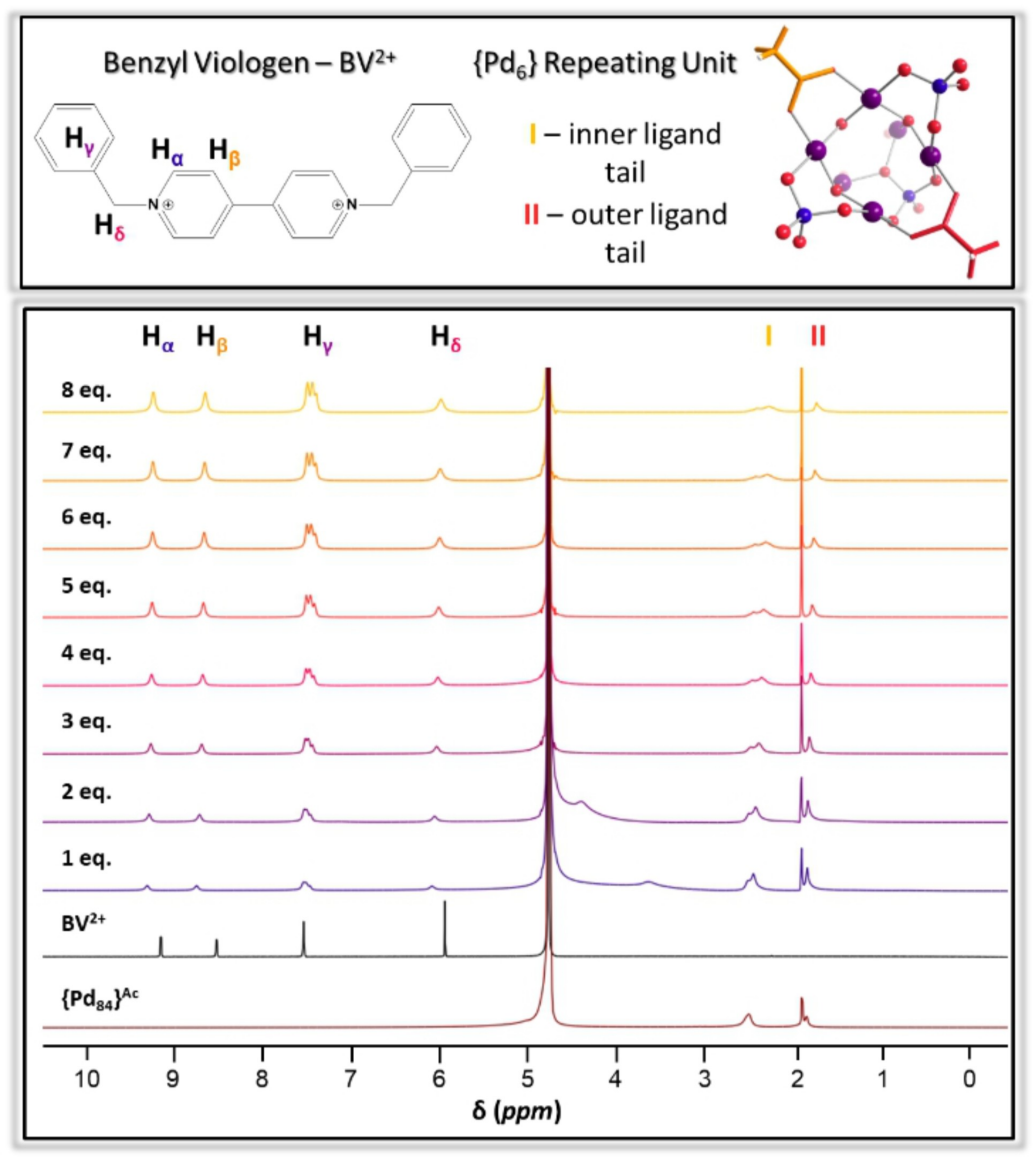
Top left; benzyl viologen structure with ^1^H NMR assignments, top right; a single {Pd_6_} repeating unit highlighting the inner and outer acetate ligands in yellow and red, respectively (purple; Pd, blue; P, red; O). Bottom; ^1^H NMR titration of 1 eq. {Pd_84_}^Ac^ and 1–8 eq. BV^2+^, (600 MHz, D_2_O, 298 K).

The ^1^H NMR titration experiment involving sequential addition of 0.18 mM solution of BV^2+^ in D_2_O to a 0.18 mM solution of {Pd_84_}^Ac^ in D_2_O shows the formation of a large supramolecular host‐guest complex (Figure 2). Adding a single equivalent of BV^2+^ to a 0.18 mM solution of {Pd_84_}^Ac^ led to notable and significant changes in the ^1^H NMR spectrum. All guest peaks shift downfield and undergo considerable broadening whilst the host experiences slight upfield shifting. The most significant chemical shift of BV^2+^ occurs for H_**α**_ and H_**β**_, Δ 0.17 ppm and Δ 0.23 ppm, respectively from their free position. Inner acetate ligand −CH_3_, **I**, resonates at 2.53 ppm when {Pd_84_}^Ac^ exists alone in solution, and moves 0.06 ppm upfield when one equivalent of BV^2+^ is added. Outer acetate ligand −CH_3_, **II**, resonates at 1.87 ppm when the polyoxopalladate is alone in solution but shifts to 1.84 ppm on addition of a single equivalent of guest. This chemical shifting process is well‐established within host‐guest chemistry and arises from changes in the shielding of nuclei which is dictated by electronegativity, magnetic anisotropy and hydrogen bonding.[^47^, ^48^, ^49^, ^50^] Broadening of NMR resonances has similar causation to peak shifting but is also impacted by exchange processes between free and bound molecules relative to the NMR timescale. The interaction between {Pd_84_}^Ac^ and BV^2+^ involves a fast exchange regime of the free BV^2+^ molecules and the bound BV^2+^ molecules resulting in a single set of ^1^H NMR peaks. Downfield shifting in ^1^H NMR is representative of a nucleus experiencing a decrease in shielding from the external magnetic field inferring its existence in an environment where the local electron density has been stripped away by an electro‐positive component. Upfield shifting signifies the opposite behaviour. These two phenomena occurring in tandem is strongly indicative of a host‐guest interaction if the two components experience opposing shielding behaviour. We hypothesise that the BV^2+ 1^H NMR resonances are downfield shifted due to the electro‐positive environment created by the {Pd_6_} building block structure. Correspondingly the inner acetate ligand −CH_3_ resonance shifts slightly upfield implying an increase in electron density caused by the addition of aromatic and π‐system containing BV^2+^ into the symmetrical pockets. The inner acetate resonance, **I**, also exhibits a new peak profile when BV^2+^ is added to the solution compared to when the nano‐wheel exists alone in solution. This is further evidence of a change in electronic and chemical environment. We believe there is a system of positive cooperativity occurring whereby the inclusion of one BV^2+^ guest promotes the inclusion of another. On addition of eight equivalents of BV^2+^ a small amount of red‐orange precipitate begins to appear. We hypothesise that this is the supramolecular complex ({Pd_84_}^Ac^@7BV^2+^) in the solid state. ESI‐MS of this precipitate, shown in Figure S10 & Table S12, shows a trend in charge:guest ratio which allows us to suggest that this precipitate represents a {Pd_84_} : BV stoichiometry of 1 : 8. This implies that after encapsulation of seven guests the 8^th^ equivalent of BV replaces an exo bound cation, initiating precipitation of the complex.

**Figure 3:**
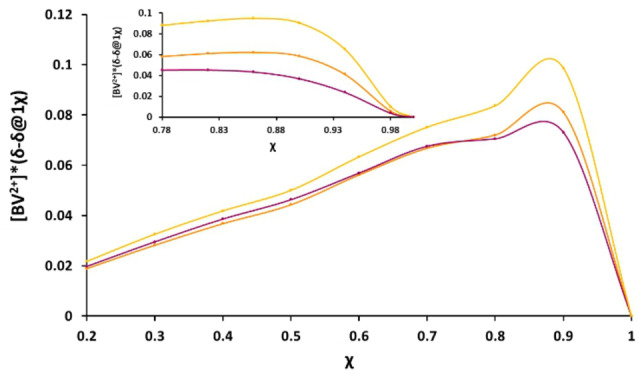
Method of continuous variation plot for BV^2+^ with {Pd_84_}^Ac^ obtained using ^1^H NMR data and using parameters from Table S3, S4 and S5. Yellow; H_β_, orange; H_α_, purple; H_δ_. Data was not obtained at 0.1 mole fraction due to low concentrations. Inset: method of continuous variation plot for BV^2+^ with {Pd_84_}^Ac^ obtained using ^1^H NMR data and using parameters from Table S8, S9 and S10. Yellow; H_β_, orange; H_α_, purple; H_δ_.

**Figure 4:**
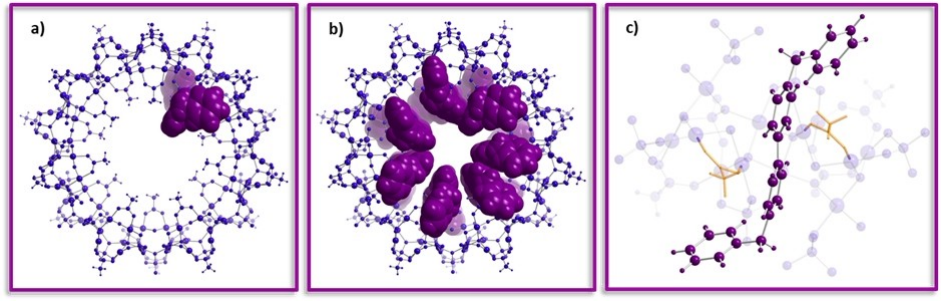
Molecular models showing a) a single BV^2+^ guest sitting in the hydrophobic pocket between two {Pd_6_} units; b) seven BV^2+^ guests within the cavity of the Pd_84_^Ac^ wheel; c) view from inside the cavity in stick and ball frame. Blue; {Pd_84_}^Ac^ framework, yellow; inner acetate ligands and purple; benzyl viologen guest.

The method of continuous variation was used to evaluate the binding stoichiometry between {Pd_84_}^Ac^ and benzyl viologen. During complexation the chemical environment of BV^2+^ protons changes, which has consequences on the chemical shift (Δδ) of each proton. In this system the guest experiences an overall de‐shielding effect with H_α_, H_β_ and H_γ_ moving downfield whilst H_δ_ is unchanged. The change in chemical shift was plotted for each guest proton, separately, and is found in Figure 3 (tables of the data used to obtain the continuous variation plot can be found on pages 12–17 of the Supporting Information). The total concentrations of both host and guest remained constant and subsequently the mole fraction, χ, was increased from 0 to 1. The maximum point on the plot of change in chemical shift (Δδ) versus the mole fraction (χ) provides the binding event stoichiometry by following Equation S2. A binding ratio of seven guest molecules to a single host represented the formation of a highly unexpected and unusual supramolecular system. To validate this, a further experiment was carried out for mole fractions 0.78–1.00, this data can be seen from Figure S7. The use of the continuous variation method has been a source of much controversy due to the limitations that occur when more than one species is present.^[51]^ However, if positive cooperativity is occurring (which we hypothesise in this system) then the method of continuous variation is reliable, according to Thordarson et al.^[51]^ To investigate the possibility of the inclusion of seven BV^2+^ guest species interacting with {Pd_84_}^Ac^ molecular visualisation was completed using WebLab ViewerLite 3.7, Figure 4. The visualised model indicates whether the molecules can fit based on their shape and size and highlights possible sites of interaction *without* computational optimisation or energy minimisation. These visualisation models suggest it is possible for seven BV^2+^ molecules to sit inside the hydrophobic cavity of {Pd_84_}^Ac^, each being held in one of the pockets by interaction of the pyridinium protons, H_α_ H_β_, and the inner ligand of the host, **I**. The benzyl rings (associated protons denoted H_γ_) at either end of the guest flank and wrap around the torus of the nano‐wheel. This is likely aided by the flexible sp^3^ hybridised methylene, H_δ,_ which links these rings to the central, rigid and cationic viologen platform.

**Figure 5:**
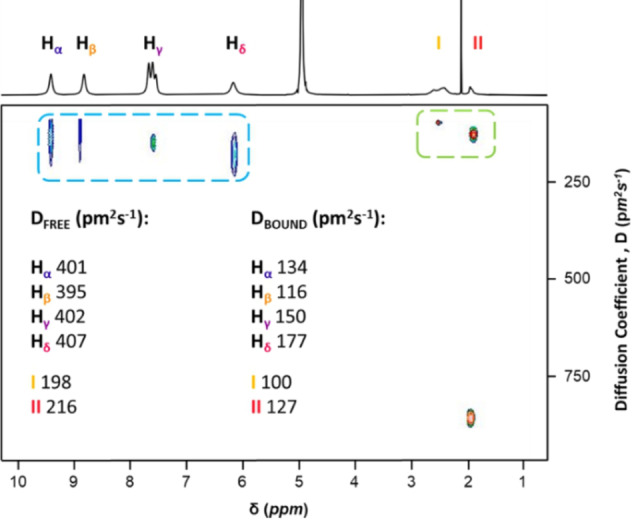
2D ^1^H DOSY NMR plot of 1 eq. {Pd_84_}^Ac^ and 7 eq. BV^2+^ in D_2_O (d20; 50 ms, p30; 1400 μs). Bound {Pd_84_}^Ac^ carboxylate ligands, I and II, highlighted in green dashed box and bound BV^2+^ guest highlighted in blue dashed box. Diffusion coefficients were calculated from the DOSY plot using Dynamics Centre.^[57]^ The peak at 1.86 ppm results from free carboxylate ligand so the diffusion coefficient is not shown. (600 MHz, D_2_O, 298 K).

2D ^1^H DOSY NMR is used to provide information about the tumbling rate of molecules in solution. It is a well‐established method in host‐guest chemistry to highlight differences in diffusion rates when guest species are bound to a host molecule compared to that of when it is moving freely.[^52^, ^53^, ^54^, ^55^, ^56^] Figure 5 shows that BV^2+^ guest and {Pd_84_}^Ac^ host tumble at an average rate of 401 pm^2^ s^−1^ and 207 pm^2^ s^−1^, respectively when they are alone in solution. In a 1 : 7 solution of the two components there is a significant decrease in the average diffusion coefficients for BV^2+^ to 144 pm^2^ s^−1^ and {Pd_84_}^Ac^ to 113 pm^2^ s^−1^. This decrease in rate of tumbling is evidence to show that the BV^2+^ is interacting with another component which slows its motion through solution. The similarity in diffusion coefficient for both host and guest components when they are together in solution suggests that they are bound together and have formed a supramolecular system. It is interesting to note that there is a slight variation in diffusion coefficient for the protons on BV^2+^, corroborating nicely with the molecular visualisation data. H_**α**_ and H_**β**_ are tightly bound within the internal pockets of the POP, interacting strongly with the methyl groups on internal acetate ligands. These two protons have smaller diffusion values than H_**γ**_ and H_**δ**_ meaning they are moving slower through solution. The methylene H_**δ**_ protons are bonded to a flexible sp^3^ carbon atom and so their motion is likely to be marginally faster. Benzylic protons, H_**γ**_, sit exposed to the outer environment and therefore experience faster motion, and a larger diffusion coefficient.

**Figure 6:**
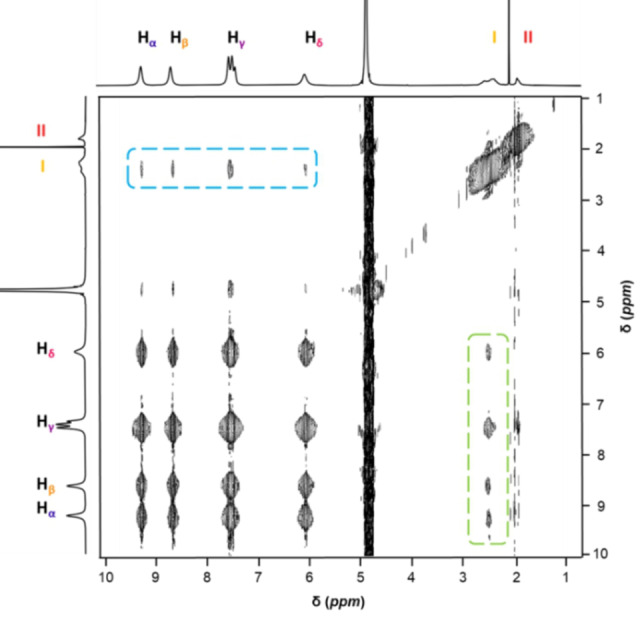
^1^H‐^1^H NOESY NMR spectrum of 1 eq. {Pd_84_}^Ac^ and 7 eq. BV^2+^ (d8; 600 ms). Cross peaks relating to coupling of BV^2+^ guest to {Pd_84_}^Ac^ host highlighted in blue dashed box and those relating to {Pd_84_}^Ac^ inner −CH_3_ ligand coupling to BV^2+^ guest highlighted in dashed green box. (600 MHz, D_2_O, 298 K).

^1^H‐^1^H NOESY NMR is used to determine through‐space interactions occurring between host and guest on complexation. Couplings from host to guest, and guest to host were observed between inner ligand −CH_3_ of {Pd_84_}^Ac^ and all four guest resonances; H_**α**_, H_**β**_ and H_**γ**_ and H_**δ**_, Figure 6. No coupling was seen between any BV^2+^ resonances and the outer ligand −CH_3_ group, **II**. It is clear from the ^1^H NMR titration data (and molecular visualisation) that guests interact exclusively within the cavity of {Pd_84_}^Ac^ and not on the outer surface, therefore couplings to outer ligand −CH_3_ of{Pd_84_}^Ac^, **II**, are not expected.

**Figure 7:**
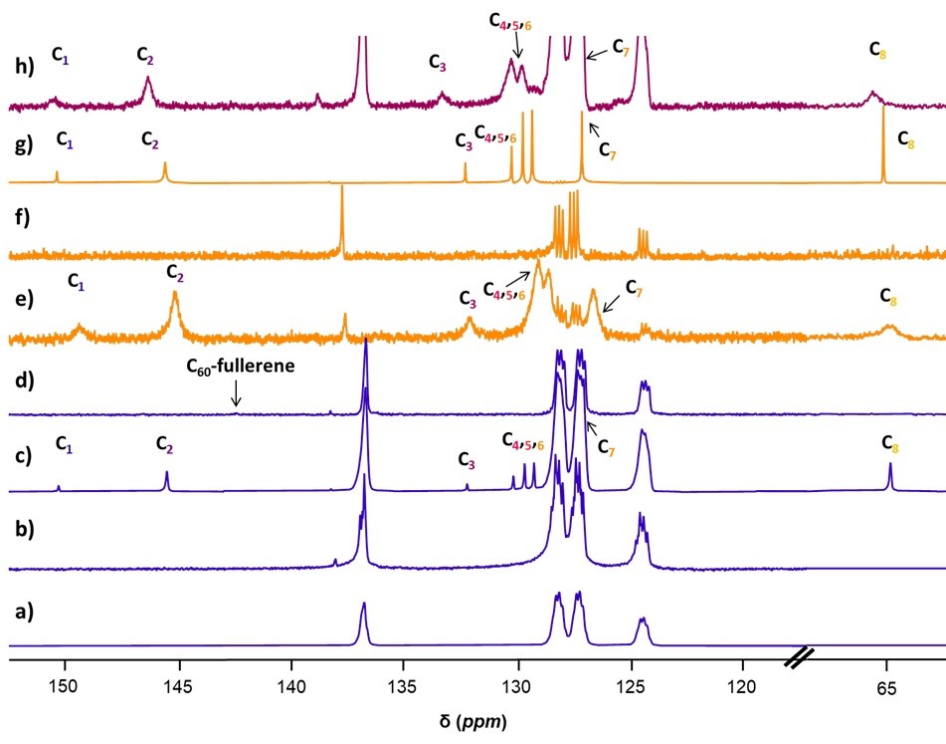
^13^C NMR spectrum of a) 500 μL D_2_O and 200 μL toluene‐d_8_; b) 1 eq. {Pd_84_}^Ac^ in 500 μL D_2_O and 200 μL toluene‐d_8_; c) 7 eq. BV^2+^ in 500 μL D_2_O and 200 μL toluene‐d_8_; d) 1 eq. C_60_‐fullerene in 500 μL D_2_O and 200 μL toluene‐d_8_; e) {Pd_84_}^Ac^ and 7 eq. BV^2+^ in 500 μL D_2_O and 200 μL toluene‐d_8_; f) {Pd_84_}^Ac^ and 1 eq. C_60_‐fullerene in 500 μL D_2_O and 200 μL toluene‐d_8_; g) BV^2+^ and 1 eq. C_60_ in 500 uL D_2_O and 200 μL toluene‐d_8_ and h) {Pd_84_}^Ac^ and 7 eq. BV^2+^ and 1 eq. C_60_‐fullerene in 500 μL D_2_O and 200 μL toluene‐d_8_. (600 MHz, D_2_O/toluene‐d_8_, 298 K). The full stacked spectra can be found in Figure S20.

Formation of the supramolecular polyoxopalladate complex, {Pd_84_}^Ac^@*n*BV^2+^, led us to investigate the possibility of further encapsulation by another species within the cavity created by the lining of the organic guest molecules. The development of supramolecular systems which include unfunctionalised C_60_‐fullerene is of great interest due to the unique properties of the carbon allotrope, but has often been limited to the C_60_‐fullerene hosting a single atom or small molecule (endohedral complex), as opposed to another larger molecule hosting C_60_‐fullerene.[^58^, ^59^, ^60^] Water soluble systems incorporating C_60_‐fullerene are even more sought after due to their potential application in biology, but this brings synthetic challenges.^[61]^ The molecular visualisation data suggests that the cationic viologen platform sits directly inside the cavity perpendicular to the torus of the nano‐wheel, with the benzyl rings protruding outwardly. The van der Waals diameter of the cavity created by the binding of seven guests is approximately 10 Å (measured using Diamond 3.0) and the diameter of C_60_‐fullerene is 7 Å. Therefore we hypothesise that the C_60_‐fullerene would sit on the edge of this cavity created by the BV^2+^ guests as opposed to sitting directly inside the secondary cavity, Figure S22. This is more likely due to the Rebek limit of 55 % which suggests that supramolecular inclusion is only possible when the guest species is ≤55 % of the diameter of the space it is intended to occupy.^[62]^ ^13^C NMR experiments of {Pd_84_}^Ac^, BV^2+^ and C_60_‐fullerene revealed shifting and broadening associated with supramolecular complexation, Figure 7. Free C_60_‐fullerene has a strong and characteristic resonance at 143.6 ppm in toluene‐d_8_. This solvent was chosen due to the poor solubility of C_60_‐fullerene in most organic solvents, despite its similar resonance to the aromatic benzyl viologen guest species. A solvent system of 5 : 2 D_2_O : toluene‐d_8_ was employed to aid solvation of the C_60_‐fullerene and because the polyoxopalladate is only soluble in aqueous solution. ^1^H‐^13^C HSQC NMR was carried out on seven equivalents of BV^2+^ in this solvent system to show that the peaks are distinguishable from one another, Figure S14. ^13^C NMR experiments were carried out on the three‐component system as well as a variety of appropriate controls and all spectra were calibrated in alignment with toluene‐d_8_ solvent peaks. The inner and outer acetate ligands of {Pd_84_}^Ac^ have two associated resonances for the carboxylate and methyl components. The inner ligand resonates at 187.4 ppm and 23.3 ppm, respectively, whilst the outer ligand resonates at 185.9 ppm and 22.5 ppm, respectively as shown in Figure S20b. On addition of BV^2+^ or C_60‐_fullerene to the polyoxopalladate there is no significant change in these shifts. The benzyl viologen guest species (carbon assignments in Figure S18) has eight distinct peaks in the spectrum‐ 150.4 ppm and 145.6 ppm for C_1_ and C_2_, 132.4 ppm for C_3_, 130.3 ppm, 129.8 ppm and 129.4 ppm for C_4_, C_5_ and C_6_, 127.2 ppm for C_7_ and 65.4 ppm for C_8_. The benzyl viologen sees a slight change in shift for all resonances when it is in the presence of the polyoxopalladate, Figure 7e, but is completely unchanged on addition of C_60_‐fullerene seen in Figure 7g. When the C_60_‐fullerene is added to the {Pd_84_}^Ac^ and BV^2+^ there is a change in chemical shift for BV^2+^. C_1_ is unchanged whilst C_2_ shifts downfield to 146.4 ppm (Δ 0.8 ppm) and C_3_ moves downfield to 133.3 ppm (Δ 0.9 ppm). C_4_, C_5_ and C_6_ are the carbon atoms associated with the benzyl rings on the BV^2+^ guest species, on addition of the C_60_‐fullerene to the pre‐formed supramolecular complex these resonances significantly broaden and C_6_ and C_7_ become slightly lost to the solvent peaks. However, using ^1^H‐^13^C HSQC NMR it is possible to distinguish these resonances from one another and to be sure they originate from the guest species rather than the solvent system‐Figure S15, S16 and S17. C_8_ is significantly shifted to 65.4 ppm (Δ 1.0 ppm). The binding of C_60_‐fullerene to the secondary cavity is most likely to be on the surface that is created by the benzyl rings of the guest which protrude from the cavity. Therefore, C_3_ and C_8_ are anticipated to be the most strongly affected by the addition of the C_60_‐fullerene, a hypothesis backed by the data.

## Conclusion

Work on supramolecular systems has focussed on organic macrocycles and their interactions with other molecules in organic solvents. Limitations of these systems include their lack of biological mimicry and the number of guest species that can be incorporated into them. Here we present a system with 7 guest molecules bound into symmetrical pockets of a nanoscale polyoxopalladate which is stable in water. ^1^H and ^13^C NMR chemical shifts as well as ^1^H NMR DOSY and NOESY data show the location of the interaction, definitively, to be on the internal surface of the nano‐wheel and to be driven by the hydrophobic effect and electrostatic forces between the sub‐units of the anionic polyoxopalladate and the cationic guest. Encapsulation of unfunctionalised C_60_‐fullerene inside this supramolecular complex was confirmed, similarly, by ^13^C NMR. This represents an interesting and biologically relevant system for potential application in biomedicine and catalysis.

## Conflict of interest

The authors declare no conflict of interest.

1

## Supporting information

As a service to our authors and readers, this journal provides supporting information supplied by the authors. Such materials are peer reviewed and may be re‐organized for online delivery, but are not copy‐edited or typeset. Technical support issues arising from supporting information (other than missing files) should be addressed to the authors.

Supporting Information

## Data Availability

The data that support the findings of this study are available in the supplementary material of this article.
