# Supplementary material for: Water‐soluble Self‐assembled {Pd84}Ac Polyoxopalladate Nano‐wheel as a Supramolecular Host
Source: Angew Chem Int Ed Engl. 2022 Nov 30;62(1):e202214203. doi: 10.1002/anie.202214203 (PMC10100005; doi:10.1002/anie.202214203)
Supplement: Supplementary file 1 — Supporting Information [file ANIE-62-0-s001.pdf]

Angewandte Chemie
Eine Zeitschrift der Gesellschaft Deutscher Chemiker

Supporting Information

Water-soluble Self-assembled $\{Pd_{84}\}^{Ac}$ Polyoxopalladate Nano-wheel as a Supramolecular Host

*Z. L. Sinclair, N. L. Bell, J. R. Bame, D.-L. Long, L. Cronin**

Supporting Information

Contents

1 Materials and Instrumentation 2
1.1 Materials 2
1.2 pH Measurements 2
1.3 Nuclear Magnetic Resonance Spectroscopy 2
2 Investigation into Reproduction Issues of $\{Pd_{84}\}^{Ac}$ 2
2.1 Palladium Precursor 2
2.2 Buffer Solution – Na_2HPO_4 – NaH_2PO_4 3
2.3 Temperature 5
2.4 Post-Reaction Filtration Method 5
2.5 Crystallisation Method 6
2.6 Crystal Isolation 6
2.7 Optimised Synthesis of $\{Pd_{84}\}^{Ac}$ 6
3 General Procedures for NMR Experiments 8
3.1 ^{1}H NMR Titration 8
3.1.1 Stock Solution of Guest 1, S_1 8
3.2 Diffusion Ordered Spectroscopy (DOSY) 8
3.3 Nuclear Overhauser Effect Spectroscopy (NOESY) 8
4 NMR Characterisation and Host-Guest Behaviour Analysis 9
4.1 1-Dimensional Data 9
4.1.1 ^{1}H NMR 9
4.1.2 Method of Continuous Variation 11
4.2 Mass Spectrometry 17
4.3 2-Dimensional NMR Data 18
4.3.1 Diffusion Ordered Spectroscopy (DOSY) 18
4.3.2 ^{1}H-^{13}C HSQC NMR Data 19
4.4 ^{13}C NMR 25
4.5 Experimental Procedures for ^{13}C NMR Work on 3-Component System 28
4.6 ^{1}H-^{1}H NOESY NMR Data 29
4.7 Molecular Visualisation of 3-Component System 30
References 31

1 Materials and Instrumentation

1.1 Materials

All reagents and solvents were purchased from Sigma-Aldrich Chemical Company Ltd., Alfa Aesar, and Tokyo Chemical Industries. Materials were used without further purification.

1.2 pH Measurements

Measurements were taken on a Hanna Instruments HI-2210-02 Bench Top pH Meter with pH electrode (HI 1131B) and temperature probe (HI 7662).

1.3 Nuclear Magnetic Resonance Spectroscopy

^{1}H, ^{13}C and associated NMR spectra were recorded on a Bruker Ascend Aeon 600 MHz NMR spectrometer. Samples were analysed in Bruker NMR tubes (5 mm diameter, 7” length).

2 Investigation into Reproduction Issues of $\{Pd_{84}\}^{Ac}$

Attempts to reproduce the synthetic procedure of $\{Pd_{84}\}^{Ac}$ were found to be difficult, and so an investigation was carried out to increase reliability and produce a more robust protocol. Despite the relative simplicity of the procedure as reported previously, there were a number of parameters that were thought to be contributing to its unreliability in yield and crystal quality.[1–3] These parameters include palladium source, sodium phosphate buffer and solution pH, stirring time, lab temperature, filtration method and crystallisation method. The following discussion analyses these parameters individually.

2.1 Palladium Precursor

$Pd(CH_3CO_2)_2$ and $Pd(NO_3)_2$ have both been used in the synthesis of $\{Pd_{84}\}^{Ac}$, with previous works from our group detailing a synthetic route to $\{Pd_{84}\}^{Gly}$ and $\{Pd_{72}\}^{Prop}$ starting from $Pd(NO_3)_2$ and adding the sodium salt of the carboxylate ligand. Since this work aimed to use $\{Pd_{84}\}^{Ac}$ as a host molecule to investigate host-guest behaviour in solution, $Pd(CH_3CO_2)_2$ was favoured over $Pd(NO_3)_2$ to decrease the number of anions present in solution.

Palladium is an expensive reagent and so a recycling process was sought to ensure that any unreacted material from $\{Pd_{84}\}^{Ac}$ syntheses was not lost. This process involved 4 stages: (i) collecting unreacted material and washings, (ii) evaporating off excess solvent by stirring to produce a concentrated slurry, (iii) converting this slurry to palladium black (Pd^0) and (iv) synthesising the acetate salt.

(i) Glassware and stir bars used in the synthesis of $\{Pd_{84}\}^{Ac}$ were thoroughly rinsed with deionised water. There is often unreacted palladium material left over as well as an insoluble side product – $\{Pd_{10}\}$. These washings were collected in a large Duran bottle until the volume was approximately 1 L.

(ii) The contents of the Duran bottle were poured into a large 3 L beaker and left to stir at room temperature until the volume had dramatically decreased to at least 25 % of its original volume (~ 250 mL) giving a black/brown mixture.

(iii) To this mixture was added 40 mL 37% HCl dropwise through a dropping funnel and then stirred for 1 hour. Zinc granules and *L*-ascorbic acid were then added, slowly, in excess, until no more *L*-ascorbic acid would dissolve. Care must be taken at this point as there is intense bubbling and the reaction is highly exothermic. The solution was left to stir overnight and was then filtered through a Buchner apparatus, leaving palladium black, which was collected.

(iv) Palladium black (6.53 g; 61.4 mmol) was added to a 1L multi-neck round bottom flask containing 300 mL glacial acetic acid. A small dropping funnel containing 5 mL 70 % HNO_3 was connected, and hot plate set to 160 °C. The system was connected to a gentle flow of N_2, and stirred until the solution began to boil at which point the hot plate was lowered to 125 °C. The dropping funnel was opened and on addition of HNO_3 to the reaction mixture brown fumes of NOx were generated. On completion of HNO_3 addition the solution was refluxed for 3 hours followed by hot filtration through a simple filter and funnel. The resulting solutions were left open in a 500 mL conical flask producing crystals overnight. Yield 2.90 g.

Initial syntheses of $\{Pd_{84}\}^{Ac}$ used commercial and recycled palladium (II) acetate. The commercial product contains trimer – $Pd_3(CH_3CO_2)_6$ – and has a powdered appearance, whilst the recycled product is highly crystalline. These crystals were analysed with single crystal X-Ray diffraction and found to be the trimer but with one acetate ligand substituted for a hydroxyl group; $Pd_3(CH_3CO_2)_5(OH)$.[4] This recycled material exhibited improved solubility and increased reliability in the production of $\{Pd_{84}\}^{Ac}$ crystals. It is hypothesised that the replacement of one acetate group by a hydroxyl group, caused by hydrolysis during the recycling process, slightly increases the solubility and contributes to the differences observed during crystallisation of $\{Pd_{84}\}^{Ac}$; difficulty in reproducing previously reported syntheses of nano-wheel.

2.2 Buffer Solution – Na_2HPO_4 – NaH_2PO_4

The previously published route to $\{Pd_{84}\}^{Ac}$ states the use of a 0.15 M solution of Na_2HPO_4-NaH_2PO_4 is prepared and the pH adjusted to 6.90 using NaOH. However, with accurate calculation of masses, weighing of both components and the use of a volumetric flask, no pH adjustment should be required according to the Henderson-Hasselbach equation. Despite this, it was consistently noted that several drops of 1 M NaOH or 1 M H_3PO_4 were required to reach pH 6.90. This led to the belief that the hydration states of the sodium phosphate salts had not been correctly taken into account in the original reporting of the procedure along. This difference in hydration state could result in incorrect masses of salts being measured altering the concentration of the solution. To make up a 0.15 M buffer solution of NaH_2PO_4-Na_2HPO_4 0.0573 moles of the conjugate acid, NaH_2PO_4, and 0.0926 moles of the conjugate base, Na_2HPO_4, are required. Comparing the relative masses of the hydrous and anhydrous forms table S1 shows that a significant difference exists in the masses.

Conjugate Acid	$NaH_2PO_4.2H_2O$	NaH_2PO_4
Molar Mass	156.01 g	119.98 g
Mass Weighed	8.94 g	6.87 g
**Conjugate Base**	**$Na_2HPO_4.7H_2O$**	**Na_2HPO_4**
Molar Mass	268.07 g	141.96 g
Mass Weighed	24.82 g	13.15 g

Table S1: Hydrous and anhydrous forms of both conjugate acid and conjugate base components used in the 0.15 M sodium phosphate buffer solution using previously reported molarities and corresponding masses.

Since the chemicals were stored in a cupboard exposed to the air it was deemed highly unlikely that the anhydrous sodium phosphate salts would be anhydrous. Their appearance was slightly clumpy and off-white as opposed to the highly crystalline white material that would be expected. Fresh batches of hydrous forms (monobasic dihydrate and dibasic heptahydrate) sodium phosphate were ordered and buffer solution made up using masses quoted in table S1. Despite this change, pH alteration was still required.

Further investigation led us to look at the calculations used to determine the molarities of both components in relation to the Henderson-Hasselbach equation 1 below.

$$(\mathbf{1})\ \mathrm{pH} = \mathrm{pKa} + \log_{10}\left(\frac{\mathrm{base}}{\mathrm{acid}}\right)$$

To work out the molarities of the components required to make a 0.15 M solution at pH 6.90 the statements below are true.

$$\text{(i) base} + \text{acid} = 0.15$$

$$\text{(ii) base} = 0.15 - \text{acid}$$

$$\text{(iii)}\ \left(\frac{\text{base}}{\text{acid}}\right) = 0.4898$$

Using these statements, the molarities the components can be accurately calculated by first substituting (ii) into (iii), giving the molarity of acid needed.

$$\text{(iv)}\ \frac{0.15 - \text{acid}}{\text{acid}} = 0.4898$$

$$\mathbf{(v)\ acid = 0.1007\ M}$$

Then taking (v) and substituting into (ii) we obtain the molarity of base needed.

$$\text{(vi) base} = 0.15 - 0.1007$$

$$\mathbf{(vii)\ base = 0.0493\ M}$$

If these molarities are used to make a 0.15 M sodium phosphate buffer solution then the pH should be exactly 6.90.

$$(\mathbf{1})\ \text{pH} = \text{pKa} + \log_{10}\left(\frac{\text{base}}{\text{acid}}\right)$$

$$\text{pH} = 7.21 + \log_{10}\left(\frac{0.0493}{0.1007}\right)$$

$$\text{pH} = 7.21 + (-0.31)$$

$$\text{pH} = 6.90$$

Taking into account these investigations of phosphate source and pH an optimised buffer solution work-up is detailed below.

"$Na_2HPO_4.7H_2O$ (13.21 g; 49.3 mmol) and $NaH_2PO_4.2H_2O$ (15.71 g; 100.7 mmol) were added to a 1 L volumetric flask and, under stirring, 700 mL deionised water was added. The pH was monitored whilst adding deionised water to make the solution up to 1 L, and at this final volume was measured to be 6.90, without requiring adjustment."

2.3 Temperature

The temperature of the reaction and the crystallisation were considered. The temperature of the reaction is not explicitly stated, but as $\{Pd_{84}\}^{Ac}$ was first discovered in this lab it was known to be a room temperature reaction. However it is a recognised fact that polyoxometalate synthesis and room temperature reactions don't go hand in hand and the time of year is often cited as a factor in yield, material quality and reaction success. With this in mind the temperature of the lab and the time of year were carefully considered. It was noted that there were substantial fluctuations in the lab temperature due to issues with the air-conditioning and the investigation was carried out during a period of significantly high outdoor temperatures making the lab unusually warm and humid. The air-conditioning unit was found to be inconsistent in its daily temperature readings and also varied depending on the time of day. This issue was resolved and the lab temperature was found to be consistently 18°C, regardless of outside temperature.

2.4 Post-Reaction Filtration Method

Once the reaction is complete the deep red solution is to be gravity filtered. The filter paper pore size is not specified in previous synthetic procedures but was deemed to be important due to the unreacted material and unwanted side-products. Whatman grade 1 filter papers with a pore size of 11 µm were commonly used. Using these filter papers initially seemed to remove all small particulate matter and leave a clear deep red solution however in the early days of the crystallisation process large quantities of the $\{Pd_{10}\}$ side-product was being produced. This means that there is less $\{PdO_4\}$

available for the desired final product. It was hypothesised that the pore size of this filter paper was not small enough to remove all the unreacted matter, leading to secondary nucleation sites being available to the {Pd_{10}} cluster and allowing it to crash out quickly. Whatman grade 6 filter papers have a much smaller pore size of 3 µm. This means that the size of particles that are allowed to pass through this filter paper is much smaller than that of the Whatman grade 1 filter paper. The use of this filter paper resulted in a marked difference in {Pd_{10}} side-products crashing out early in the 10 day crystallisation process, meaning there was more {PdO_4} in solution to contribute to the self-assembly of the nano-wheel, {Pd_{84}}^{Ac}.

2.5 Crystallisation Method

Crystallisation is a complex process involving nucleation and crystal growth which are driven by thermodynamic and kinetic properties, and occur from super-cooling of a liquid or super-saturation of a solvent. In the case of {Pd_{84}}^{Ac} the crystallisation process arises due to the super-saturation of the solvent, water. A variety of crystallisation conditions were experimented with. Beakers containing the {Pd_{84}}^{Ac} filtrate were left covered by Parafilm, covered by Parafilm with small holes or totally uncovered and at either 18°C or 25°C. The higher temperature of 25°C was found to be far too high resulting in complete evaporation of the solution within a few days, leaving behind a thick red sludge which soon dried up. Leaving the beakers covered, partially covered or completely uncovered seemed to make little to no difference in the time for the distinctive red needle-like crystals to appear and so it was decided to go forward with beakers being totally uncovered.

2.6 Crystal Isolation

The method used to isolate the crystals is not discussed in any previous literature or theses. It was found to be incredibly difficult and time-consuming due to the fine nature of the crystals, level of super-saturation of the solution and the very small scale of the reaction (after 10 days only around 1 mL of solution remains). Traditional methods of Bucher filtration were not possible due to huge losses in transferring the crystals to the apparatus. A method using a glass pipette, glass wool and a very slow N_2 flow was devised and can be seen in the figure below. This method, although time-consuming, dramatically decreased the transfer losses moving the solution from the reaction vessel to the filtration apparatus.

2.7 Optimised Synthesis of {Pd_{84}}^{Ac}

$Pd_3(CH_3CO_2)_5(OH)$ (0.56 g; 2.5 mmol) was added to a 50 mL beaker along with 22 mL 0.15 M Na_2HPO_4-NaH_2PO_4 buffer solution at pH 6.90. The beaker was covered with parafilm and the suspension was stirred vigorously for 20 hours at room temperature. After this time the dark red solution was filtered through a Whatman grade 6 filter paper. The solution was left to crystallise, uncovered, at 18°C. After a few days, orange precipitate began to form and was removed by filtration. This was found to be {$Pd_{10}(CH_3CO_2)_{12}O_4(H_2O)_2$}. From day eight onwards, long red needle-like crystals began to form. These crystals were collected using Buchner apparatus, and left to dry overnight under vacuum. Yield: 27 % based on Pd.

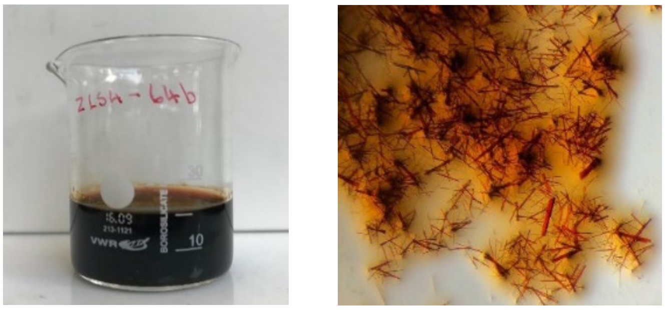

Figure S1: Left; beaker of {Pd_{84}}^{Ac} solution at day 2. Right; fine red needles of {Pd_{84}}^{Ac} through a microscope at day 10.

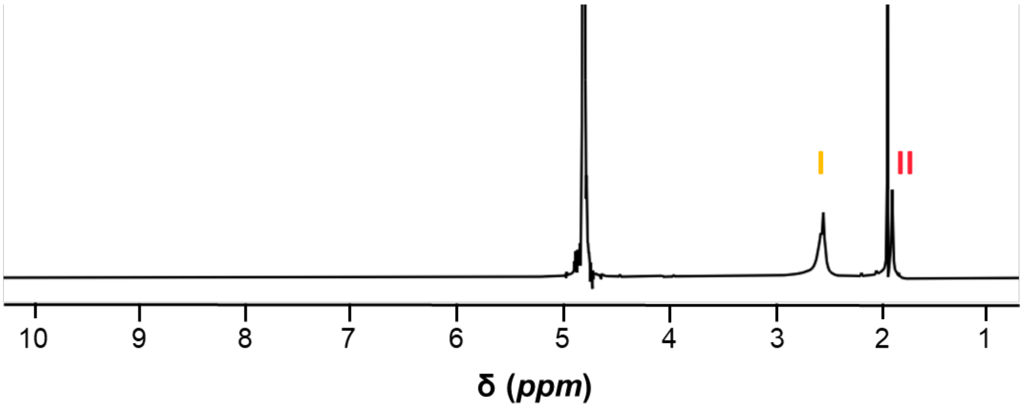

Figure S2: ^{1}H NMR of {Pd_{84}}^{Ac}. **I**; inner acetate –CH_3, **II**; outer acetate –CH_3. (600 MHz, D_2O, 298 K).

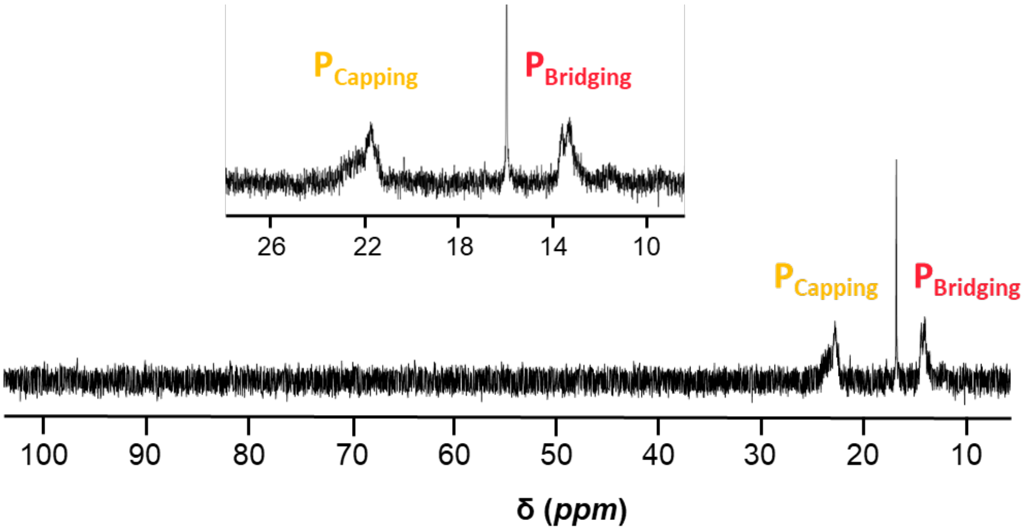

Figure S3: ^{31}P NMR of {Pd_{84}}^{Ac} (600 MHz, D_2O, 298 K).

3 General Procedures for NMR Experiments

3.1 ^{1}H NMR Titration

A Bruker NMR tube (5 mm diameter, 7" length) containing 20 mg $\{Pd_{84}\}^{Ac}$ in 400 µL D_2O (1×10^{-3} mmol; 2.48 mM) was used as the starting solution, S_0, for the titration. Stock solutions, S_n, of each guest were made up so *X* µL was equal to 1 equivalent, based on the number of moles of $\{Pd_{84}\}^{Ac}$.

3.1.1 Stock Solution of Guest 1, S_1

= 5.16 mg BV^{2+} (409.35 $gmol^{-1}$) in 240 µL D_2O

= 20 µL S_1 contains 0.43 mg BV^{2+}

= 1×10^{-3} mmol BV^{2+} in 20 µL S_1 is 1 equivalent

^{1}H NMR experiment (16 scans, AQ (acquisition time) 2 *s*, d1 (relaxation delay) 4 *s*) was carried out on S_0, then 1 equivalent of S_1 was added to S_0 and experiment run with the same acquisition parameters as for S_0. This process was repeated for sequential additions of S_n, up to and including 8 equivalents, at which point the experiment was ceased. ^{1}H NMR experiments were completed on samples containing free guest species, with a concentration of 2.5 mM and the same acquisition parameter set.

3.2 Diffusion Ordered Spectroscopy (DOSY)

A Bruker NMR tube (5 mm diameter, 7" length) containing 20 mg $\{Pd_{84}\}^{Ac}$ in 400 µL D_2O (1×10^{-3} mmol; 2.48 mM) and 1 eq. of S_1, followed by 180 µL D_2O was analysed. Optimisation of these experiments generally followed the procedure outlined in the Bruker TopSpin DOSY manual - http://sopnmr.ucsd.edu/assets/pdf/DOSY_and_Diffusion.pdf – with some variation in acquisition parameters to suit our chemical system. Important parameters include diffusion gradient length (p30; 900 µs), diffusion time (d20; 50 ms) and eddy current delay (d21; 5 ms).

3.3 Nuclear Overhauser Effect Spectroscopy (NOESY)

A Bruker NMR tube (5 mm diameter, 7" length) containing 20 mg $\{Pd_{84}\}^{Ac}$ in 400 µL D_2O (1×10^{-3} mmol; 2.48 mM) and 4 eq. of S_1 and 120 µL D_2O was analysed. Important parameters include mixing time, d8, which was set at 600 ms.

4 NMR Characterisation and Host-Guest Behaviour Analysis

4.1 1-Dimensional Data

4.1.1 ^{1}H NMR

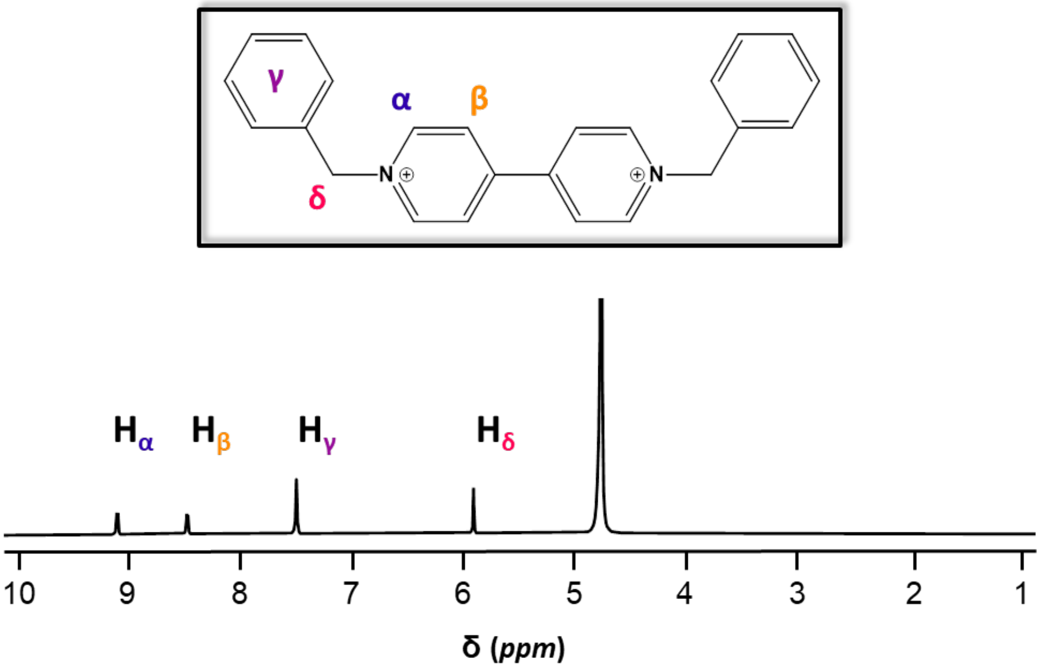

Figure S4: ^{1}H NMR of BV^{2+} (600 MHz, D_2O, 298 K).

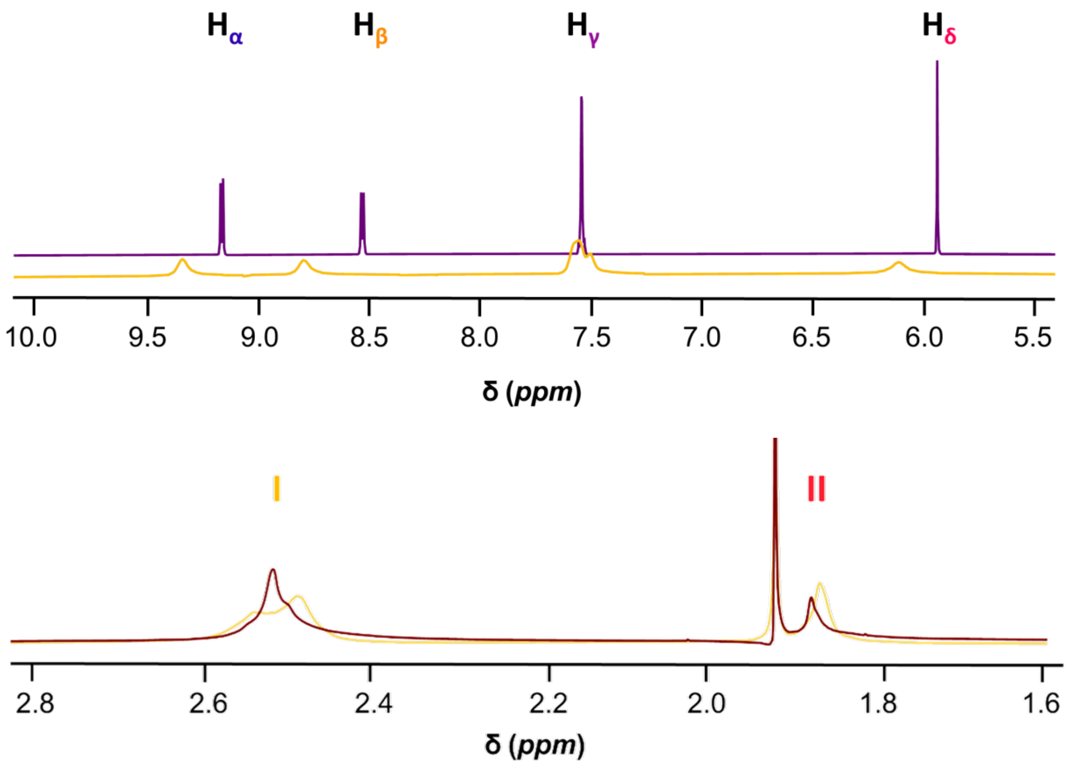

Figure S5: ^1H NMR (600 MHz, D_2O, 298 K) spectra overlay of $\{Pd_{84}\}^{Ac}$ alone (red), BV^{2+} alone (purple), and $\{Pd_{84}\}^{Ac}$ with 1 eq. BV^{2+} (yellow) showing the change in chemical shift on addition of guest to host. Δδ: $H_α$; + 0.17 ppm, $H_β$; + 0.23 ppm, $H_δ$;+ 0.15 ppm, $H_γ$; 0.00 ppm, I; - 0.05 ppm and II; - 0.01 ppm. Changes in chemical shift are relative to the free position, positive values indicate downfield shift, negative values indicate upfield shift.

4.1.2 Method of Continuous Variation

Volume of $\{Pd_{84}\}^{Ac}$ (µL)	Volume of BV^{2+} (µL)	Mole Fraction of BV^{2+}, χ	Concentration (mM)	
			$\{Pd_{84}\}^{Ac}$	BV^{2+}
600	0	0	0.413	0
540	60	0.1	0.372	0.0413
480	120	0.2	0.3304	0.0826
420	180	0.3	0.2891	0.1239
360	240	0.4	0.2478	0.1652
300	300	0.5	0.2065	0.2065
240	360	0.6	0.1652	0.2478
180	420	0.7	0.1239	0.2891
120	480	0.8	0.0826	0.3304
60	540	0.9	0.0413	0.372
0	600	1	0	0.413

Table S2: Experimental parameters for continuous variation experiment. Stock solution of $\{Pd_{84}\}^{Ac}$ contained 32.5 mg material in 3900 µL D_2O. Stock solution of BV^{2+} contained 0.65 mg material in 3900 µL D_2O.

H_α			
Mole Fraction of BV^{2+}, χ	Chemical Shift, δ (ppm)	$[BV^{2+}]$ (mM)	$[BV^{2+}]$*(δ-δ@1χ)
0	0	0	0
0.1	0	0.0413	-
0.2	9.2948	0.0826	0.01886584
0.3	9.2939	0.1239	0.02818725
0.4	9.2889	0.1652	0.036757
0.5	9.2809	0.2065	0.04429425
0.6	9.293	0.2478	0.05615148
0.7	9.2974	0.2891	0.0667821
0.8	9.2844	0.3304	0.0720272
0.9	9.2844	0.372	0.081096
1	9.0664	0.413	0

Table S3: Chemical shifts of proton H_α obtained from the experiment using parameters in **Table S2** with the corresponding concentrations of guest, BV^{2+}, and the expression $[BV^{2+}]$*(δ-δ@1χ).[5,6]

H_β			
**Mole Fraction of BV^{2+}, χ**	**Chemical Shift, δ (ppm)**	**[BV^{2+}] (mM)**	**[BV^{2+}]*(δ-δ@1χ)**
0	0	0	0
0.1	0	0.0413	-
0.2	8.6962	0.0826	0.02174858
0.3	8.6957	0.1239	0.03256092
0.4	8.6862	0.1652	0.04184516
0.5	8.6752	0.2065	0.05003495
0.6	8.6886	0.2478	0.06336246
0.7	8.6929	0.2891	0.075166
0.8	8.6862	0.3304	0.08369032
0.9	8.6981	0.372	0.0986544
1	8.4329	0.413	0

Table S4: Chemical shifts of proton H_β obtained from the experiment using parameters in **Table S2** with the corresponding concentrations of guest, BV^{2+}, and the expression [BV^{2+}]*(δ-δ@1χ).[5,6]

H_δ			
**Mole Fraction of BV^{2+}, χ**	**Chemical Shift, δ (ppm)**	**[BV^{2+}] (mM)**	**[BV^{2+}]*(δ-δ@1χ)**
0	0	0	0
0.1	0	0.0413	-
0.2	6.0855	0.0826	0.01974966
0.3	6.0848	0.1239	0.02953776
0.4	6.0801	0.1652	0.03860724
0.5	6.0706	0.2065	0.0462973
0.6	6.076	0.2478	0.05689488
0.7	6.0801	0.2891	0.06756267
0.8	6.0598	0.3304	0.07050736
0.9	6.0429	0.372	0.073098
1	5.8464	0.413	0

Table S5: Chemical shifts of proton H_δ obtained from the experiment using parameters in **Table S2** with the corresponding concentrations of guest, BV^{2+}, and the expression [BV^{2+}]*(δ-δ@1χ).[5,6]

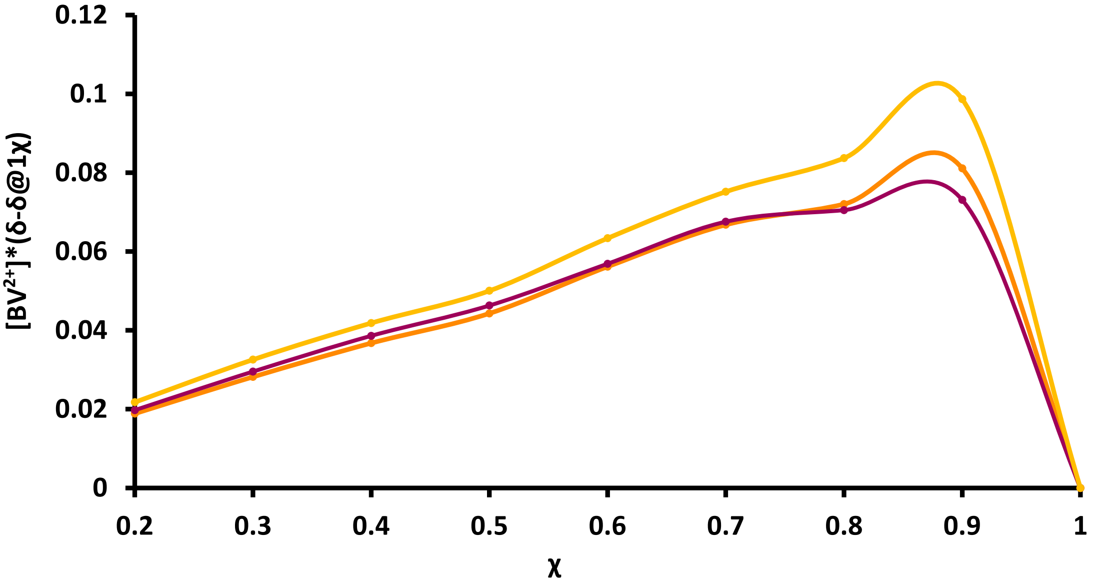

Figure S6: Method of continuous variation plot for BV^{2+} with $\{Pd_{84}\}^{Ac}$ obtained using ^{1}H NMR data and using parameters from **Table S3**, **S4** and **S5**. Yellow; H_β, orange; H_α, purple; H_δ. Data was not obtained at 0.1 mole fraction due to low concentrations.

$$(\mathbf{S2})\ \text{Binding Ratio} = \frac{\text{mole fraction at maximum}}{1.0 - \text{mole fraction maximum}}$$

Proton	Mole Fraction of BV^{2+} at Maximum, χ	Binding Ratio (H:G)	Average Binding Ratio
H_α	0.88	1:7.3	1:7.1
H_β	0.88	1:7.3	
H_δ	0.87	1:6.7	

Table S6: Maximum mole fraction values extracted from **Figure S6** for each proton and the resulting binding ratio calculated using **Equation S2**.

Volume of $\{Pd_{84}\}^{Ac}$ (μL)	Volume of BV^{2+} (μL)	Mole Fraction of BV^{2+}, χ	Concentration (mM)	
			$\{Pd_{84}\}^{Ac}$	BV^{2+}
132	468	0.78	0.09086	0.32214
108	492	0.82	0.07434	0.33866
84	516	0.86	0.05782	0.35518
60	540	0.90	0.0413	0.3717
36	564	0.94	0.02478	0.38822
12	588	0.98	0.00826	0.40474
0	600	1.00	0	0.413

Table S7: Experimental parameters for continuous variation experiment examining the region of 0.78 – 1.00 mole fraction. Stock solution of $\{Pd_{84}\}^{Ac}$ contained 32.5 mg material in 3900 μL D_2O. Stock solution of BV^{2+} contained 0.65 mg material in 3900 μL D_2O.

H_α			
Mole Fraction of BV^{2+}, χ	Chemical Shift, δ (ppm)	$[BV^{2+}]$ (mM)	$[BV^{2+}]$*(δ-δ@1χ)
0.78	9.3428	0.32214	0.058178484
0.82	9.3423	0.33866	0.060992666
0.86	9.3373	0.35518	0.062192018
0.90	9.32	0.3717	0.05865426
0.94	9.268	0.38822	0.041073676
0.98	9.1764	0.40474	0.005747308
1.00	9.1622	0.413	0
H_β			
Mole Fraction of BV^{2+}, χ	Chemical Shift, δ (ppm)	$[BV^{2+}]$ (mM)	$[BV^{2+}]$*(δ-δ@1χ)
0.78	8.8028	0.32214	0.088169718
0.82	8.8017	0.33866	0.092318716
0.86	8.7959	0.35518	0.094762024
0.90	8.7726	0.3717	0.09050895
0.94	8.697	0.38822	0.065182138
0.98	8.5543	0.40474	0.010199448
1.00	8.5291	0.413	0

Table S8: Chemical shifts of proton H_α obtained from the experiment using parameters in **Table S7** with the corresponding concentrations of guest, BV^{2+}, and the expression $[BV^{2+}]$*(δ-δ@1χ).[5,6]

Table S9: Chemical shifts of proton H_β obtained from the experiment using parameters in **Table S7** with the corresponding concentrations of guest, BV^{2+}, and the expression $[BV^{2+}]$*(δ-δ@1χ).[5,6]

H_δ			
Mole Fraction of BV^{2+}, χ	Chemical Shift, δ (ppm)	$[BV^{2+}]$ (mM)	$[BV^{2+}]*(\delta-\delta@1\chi)$
0.78	6.0821	0.32214	0.045067386
0.82	6.0756	0.33866	0.045177244
0.86	6.0641	0.35518	0.043296442
0.90	6.0414	0.3717	0.03687264
0.94	6.0033	0.38822	0.023720242
0.98	5.9515	0.40474	0.003764082
1.00	5.9422	0.413	0

Table S10: Chemical shifts of proton H_δ obtained from the experiment using parameters in **Table S7** with the corresponding concentrations of guest, BV^{2+}, and the expression $[BV^{2+}]*(\delta-\delta@1\chi)$.[5,6]

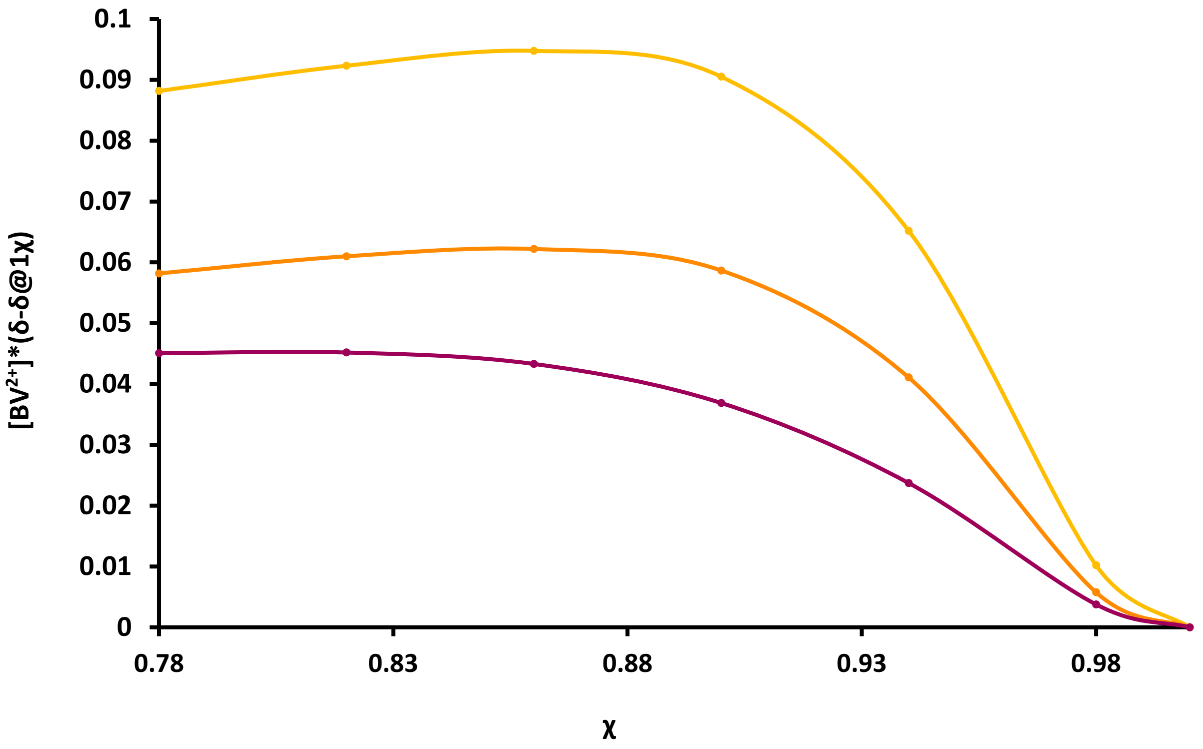

Figure S7: Method of continuous variation plot for BV^{2+} with $\{Pd_{84}\}^{Ac}$ obtained using ^{1}H NMR data and using parameters from **Table S8**, **S9** and **S10**. Yellow; H_β, orange; H_α, purple; H_δ. Data was not obtained at 0.1 mole fraction due to low concentrations.

Proton	Mole Fraction of BV^{2+} at Maximum, χ	Binding Ratio (H:G)	Average Binding Ratio
H_α	0.87	1:6.7	1:6.5
H_β	0.87	1:6.7	
H_δ	0.86	1:6.1	

Table S11: Maximum mole fraction values extracted from **Figure S7** for each proton and the resulting binding ratio calculated using **Equation S2**.

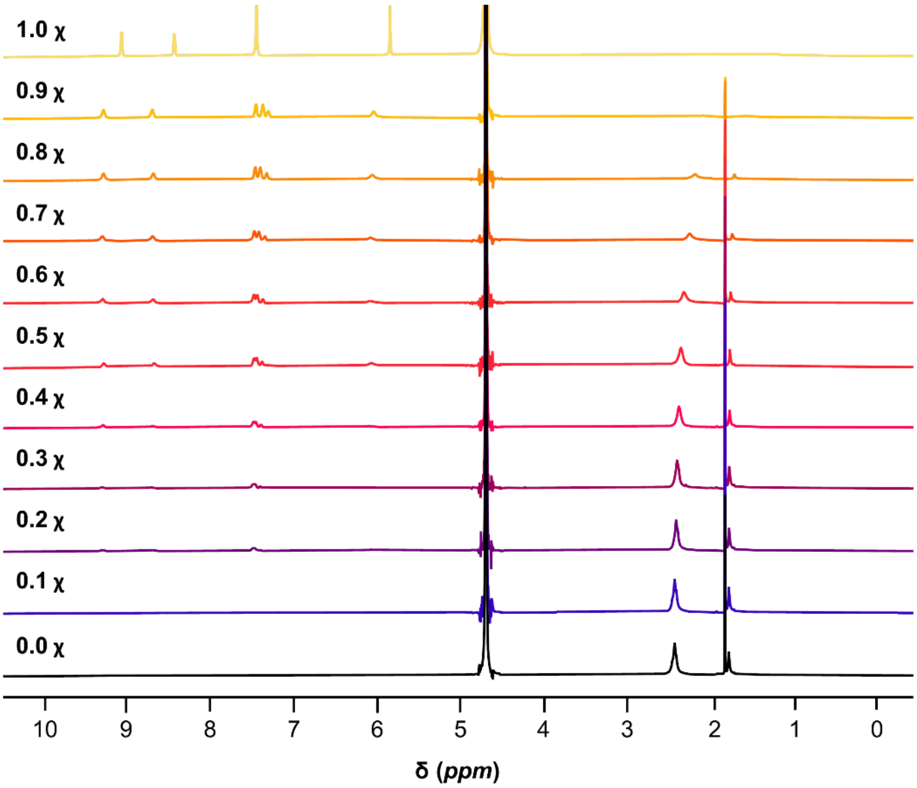

Figure S8: ^{1}H NMR stacked spectra of method of continuous variation experiment using $\{Pd_{84}\}^{Ac}$ and BV^{2+}. (600 MHz, D_2O, 298 K).

4.2 Mass Spectrometry

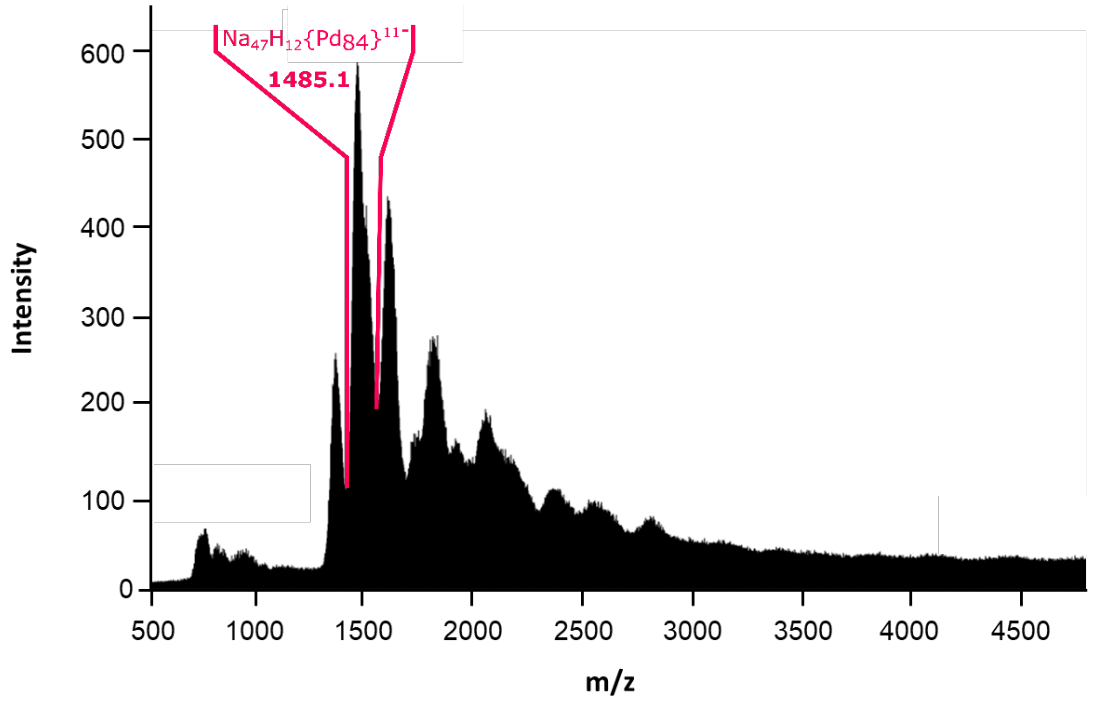

Figure S9: $\{Pd_{84}\}^{Ac}$ in water/acetonitrile mix.

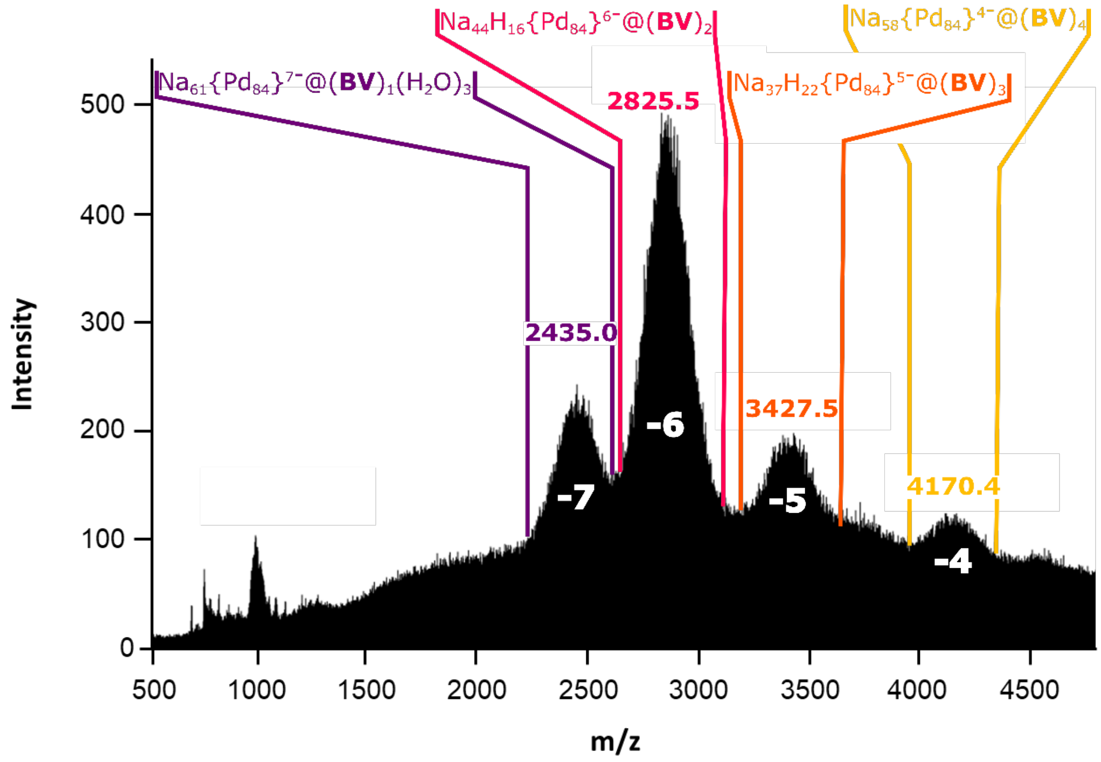

Figure S10: $\{Pd_{84}\}^{Ac}$@nBV^{2+} in water/acetonitrile mix.

Table S12: Assigned molecular ion peaks for $\{Pd_{84}\}^{Ac}$@nBV^{2+} in water/acetonitrile mix. *outside of spectral range. † Overlapping peaks.

**Assignment**	**Formula**	***z***	***m/z* (obs)**	***m/z* (calc)**
$Na_{61}\{Pd_{84}\}@(BV)_1(H_2O)_3$	$Na_{61}Pd_{84}O_{42}(OAc)_{28}(PO_4)_{42}{}^{7-}(C_{24}H_{22}N_2)_1(H_2O)_3$	−7	2435.0†	2435.1
$Na_{61}\{Pd_{84}\}@(BV)_1$	$Na_{61}Pd_{84}O_{42}(OAc)_{28}(PO_4)_{42}{}^{7-}(C_{24}H_{22}N_2)_1$	−7	2428.5†	2427.9
$Na_{44}H_{16}\{Pd_{84}\}@(BV)_2$	$Na_{44}H_{16}Pd_{84}O_{42}(OAc)_{28}(PO_4)_{42}{}^{6-}(C_{24}H_{22}N_2)_2$	−6	2825.5	2826.0
$Na_{37}H_{22}\{Pd_{84}\}@(BV)_3$	$Na_{37}H_{22}Pd_{84}O_{42}(OAc)_{28}(PO_4)_{42}{}^{5-}(C_{24}H_{22}N_2)_3$	−5	3427.5	3427.7
$NaH_{56}\{Pd_{84}\}@(BV)_4$	$NaH_{56}Pd_{84}O_{42}(OAc)_{28}(PO_4)_{42}{}^{4-}(C_{24}H_{22}N_2)_4$	−4	4170.4	4170.7
$H_{57}\{Pd_{84}\}@(BV)_5$	$NaH_{57}Pd_{84}O_{42}(OAc)_{28}(PO_4)_{42}{}^{3-}(C_{24}H_{22}N_2)_5$	−3	*	5666.9
$H_{57}\{Pd_{84}\}@(BV)_6$	$NaH_{56}Pd_{84}O_{42}(OAc)_{28}(PO_4)_{42}{}^{2-}(C_{24}H_{22}N_2)_6$	−2	*	8669.2
$H_{57}\{Pd_{84}\}@(BV)_7$	$NaH_{55}Pd_{84}O_{42}(OAc)_{28}(PO_4)_{42}{}^{1-}(C_{24}H_{22}N_2)_7$	−1	*	17676.1
$(BV)H_{54}\{Pd_{84}\}@(BV)_7$	$(BV)H_{54}Pd_{84}O_{42}(OAc)_{28}(PO_4)_{42}(C_{24}H_{22}N_2)_7$	0	*	18013.3

4.3 2-Dimensional NMR Data

4.3.1 Diffusion Ordered Spectroscopy (DOSY)

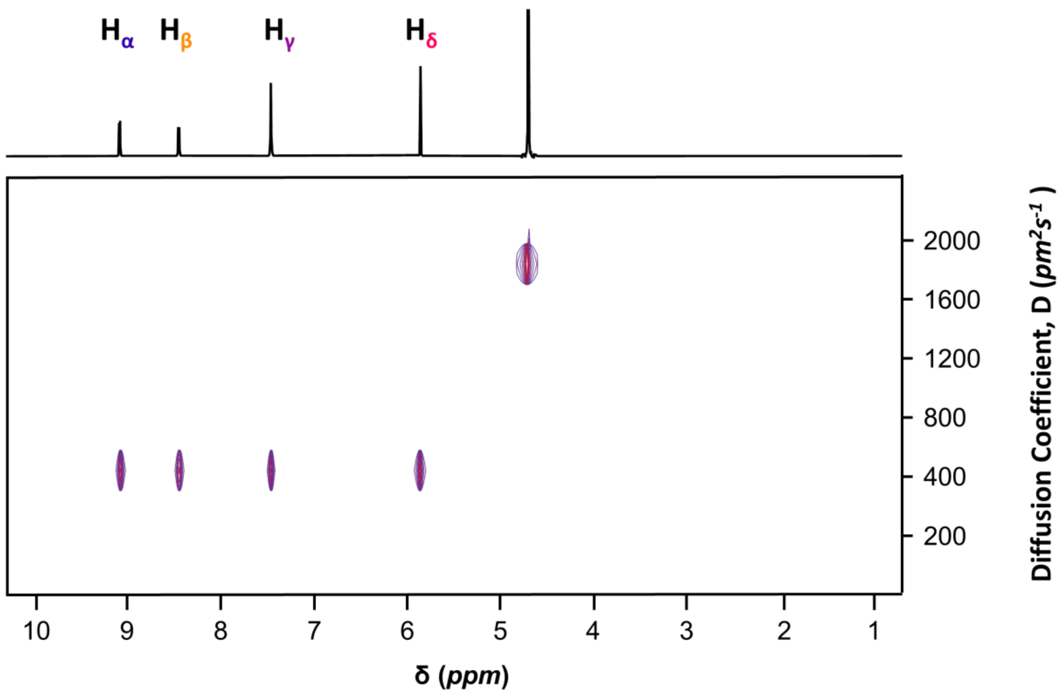

Figure S11: 2D ^{1}H DOSY NMR plot of BV^{2+} alone in D_2O. H_α; 401 pm^2s^{-1} , H_β; 395 pm^2s^{-1}, H_δ; 402 pm^2s^{-1}, H_γ; 407 pm^2s^{-1}. (600 MHz, D_2O, 298 K).

4.3.2 ^1H-^{13}C HSQC NMR Data

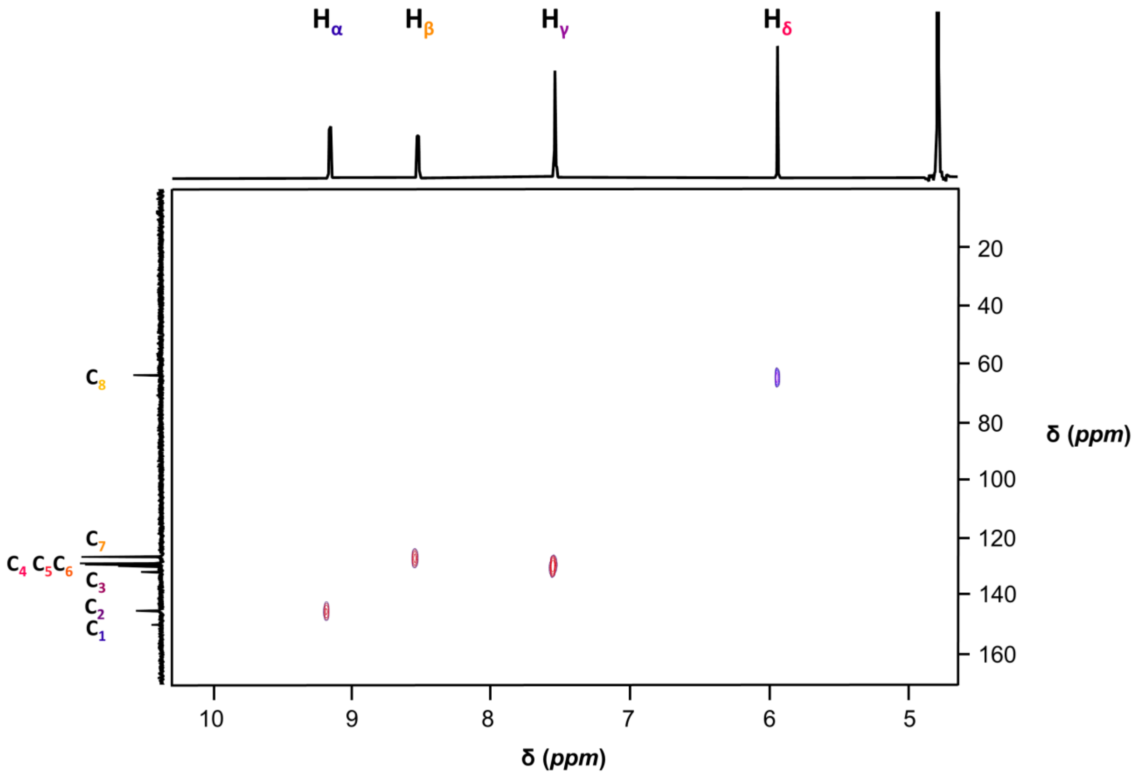

Figure S12: ^1H-^{13}C HSQC of BV^{2+} showing correlations between H_α and C_2, H_β and C_7, H_γ and C_4, C_5 and C_6 and H_δ and C_8. (600 MHz, D_2O, 298 K).

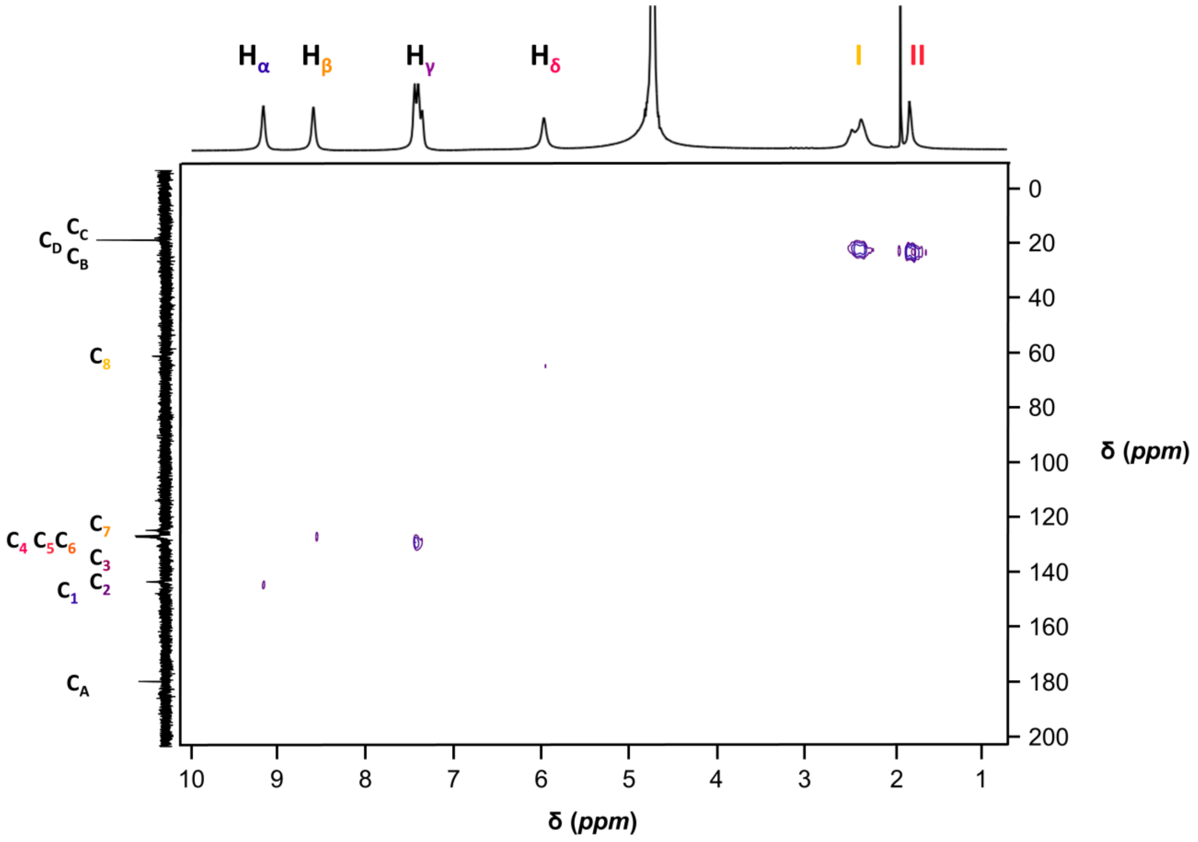

Figure S13: ^{1}H-^{13}C HSQC of $\{Pd_{84}\}^{Ac}$ and 1 eq. BV^{2+} showing correlations between H_{α} and C_2, H_{β} and C_7, H_{γ} and C_4, C_5 and C_6, H_{δ} and C_8. (600 MHz, D_2O, 298 K).

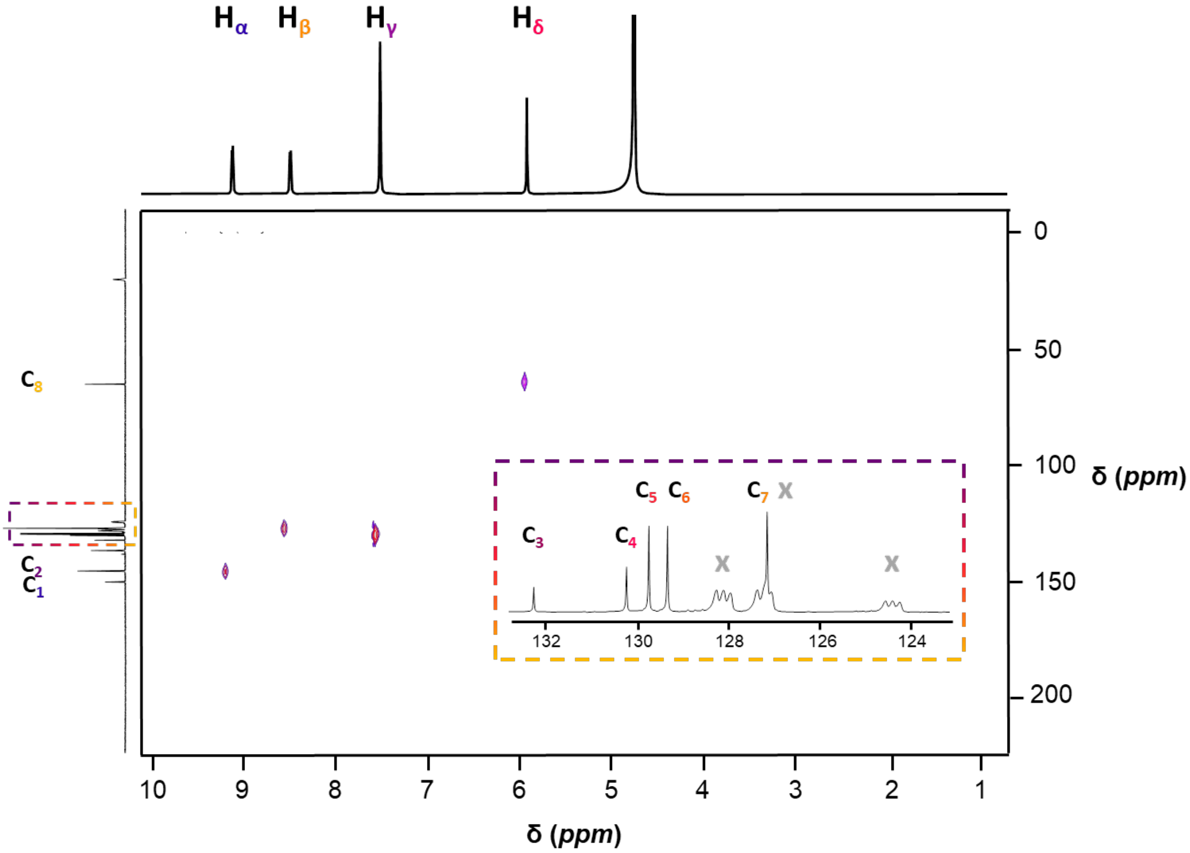

Figure S14: ^{1}H-^{13}C HSQC of BV^{2+}. Highlighted box shows that although toluene-d_8 and BV^{2+} both resonate in the aromatic region, they are distinguishable to one another through their HSQC correlations. (600 MHz, D_2O/toluene-d_8, 298 K).

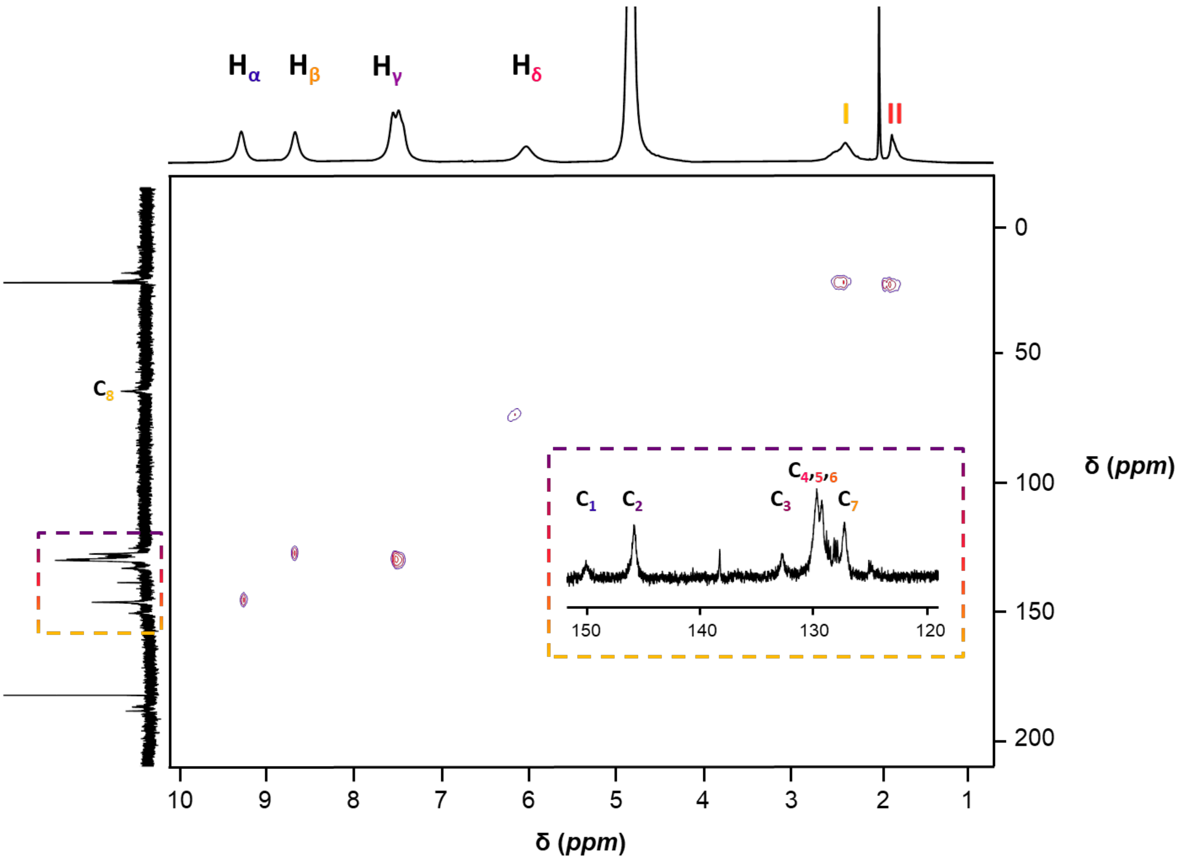

Figure S15: ^{1}H-^{13}C HSQC of $\{Pd_{84}\}^{Ac}$ and 7 eq. BV^{2+}. (600 MHz, D_2O/toluene-d_8, 298 K).

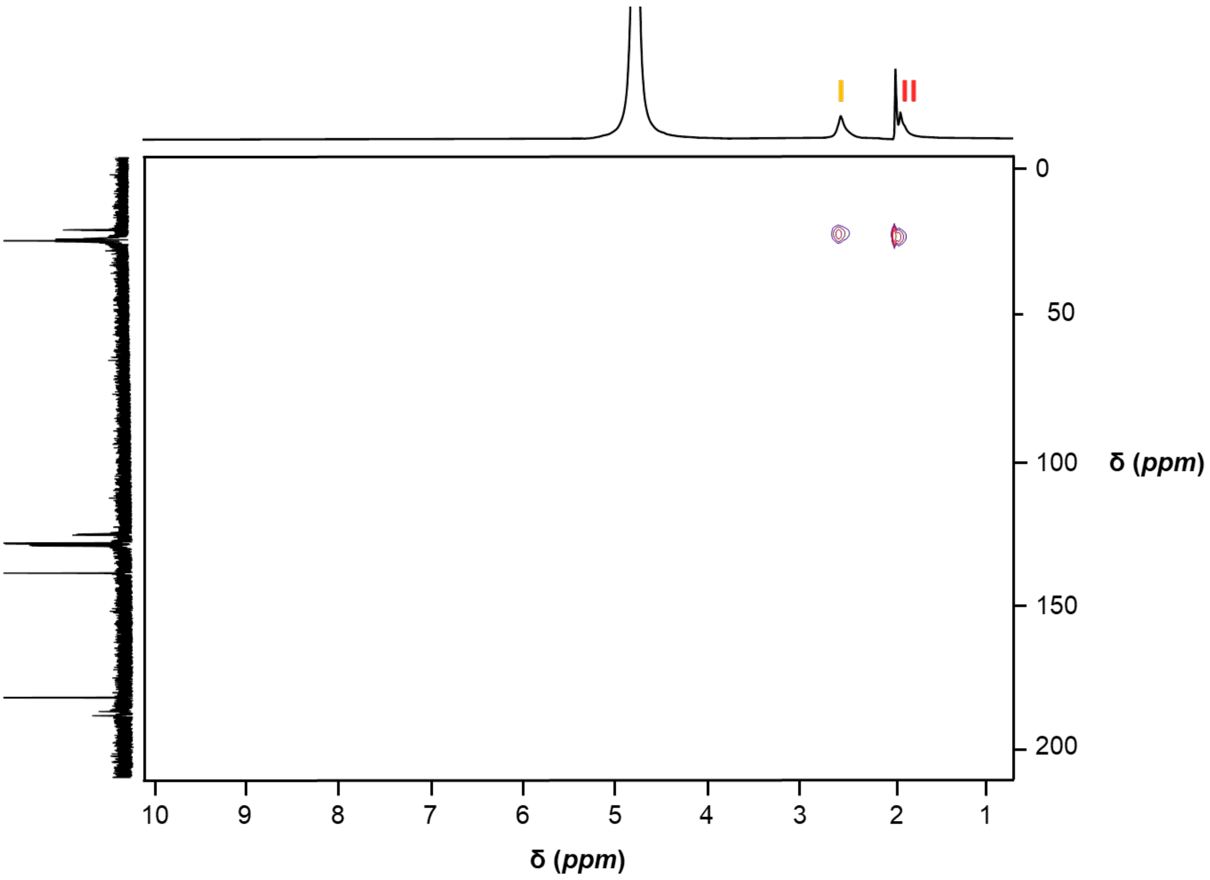

Figure S16: ^1H-^{13}C HSQC of $\{Pd_{84}\}^{Ac}$ and C_{60}-fullerene. The absence of any correlations other than of the –CH_3 of the inner and outer ligands suggests that the peaks resonating between 120 – 140 ppm do not originate from the $\{Pd_{84}\}^{Ac}$ host. (600 MHz, D_2O/toluene-d_8, 298 K).

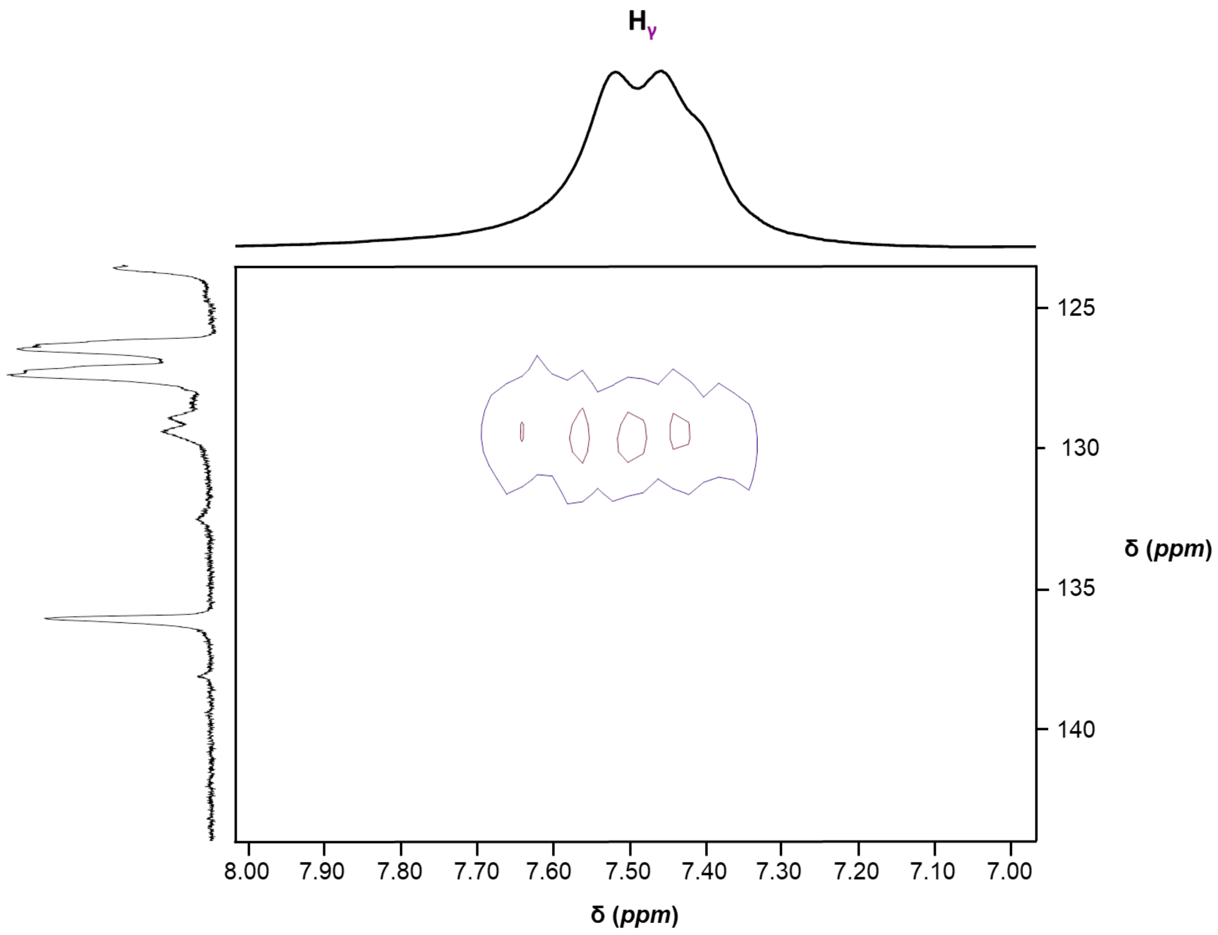

Figure S17: ^{1}H-^{13}C HSQC of $\{Pd_{84}\}^{Ac}$, 7 eq. BV^{2+} and 1 eq. C_{60}-fullerene zoomed only to show the benzyl protons, H_γ, correlate to the ^{13}C NMR spectrum. This highlights that it is possible to distinguish the benzyl carbon atoms from the toluene-d_8 carbon atoms and that the small and broad

peaks resonating at 129.9 ppm and 130.3 ppm can definitively be assigned to the guest molecule. (600 MHz, D_2O/toluene-d_8, 298 K).

4.4 ^{13}C NMR

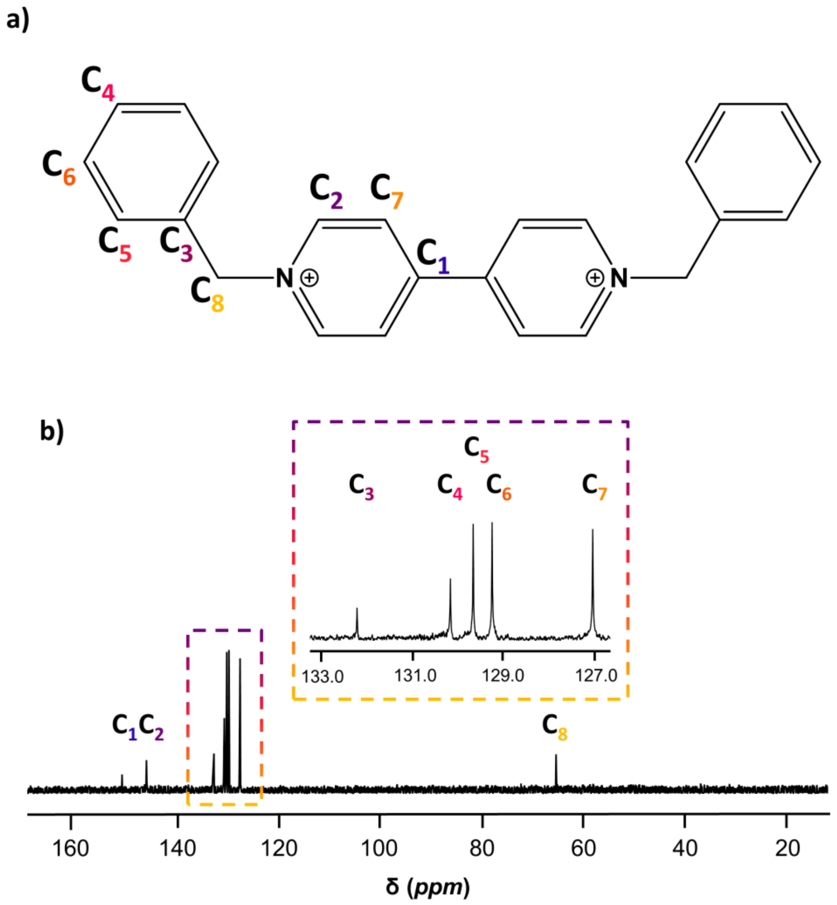

Figure S18: ^{13}C NMR of BV^{2+} (600 MHz, D_2O, 298 K).

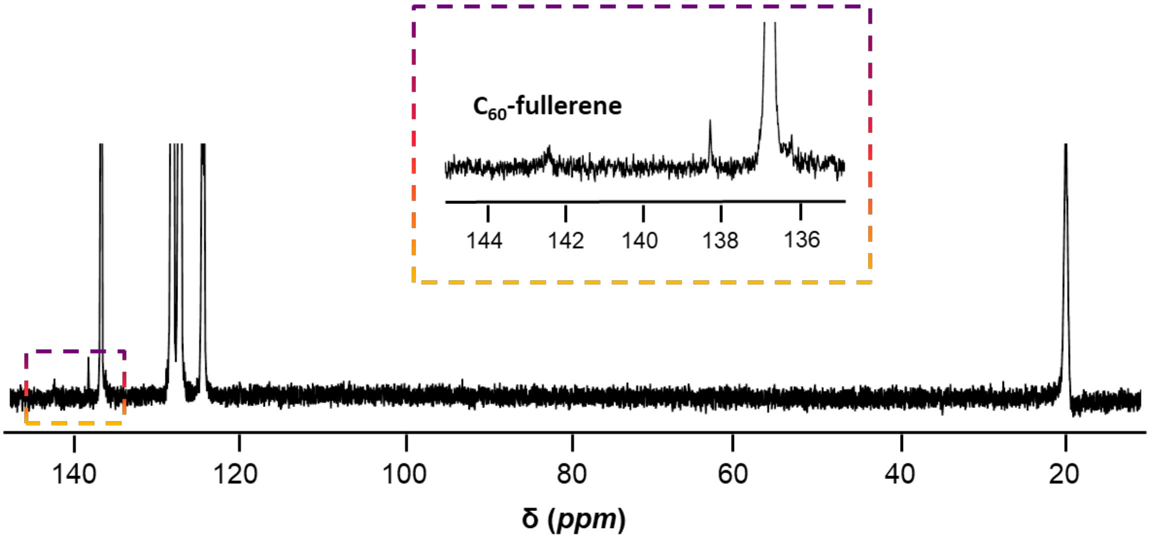

Figure S19: ^{13}C NMR of C_{60}-fullerene alone, highlighting the singlet resonance at 142.3 ppm, all other peaks are toluene-d_8.

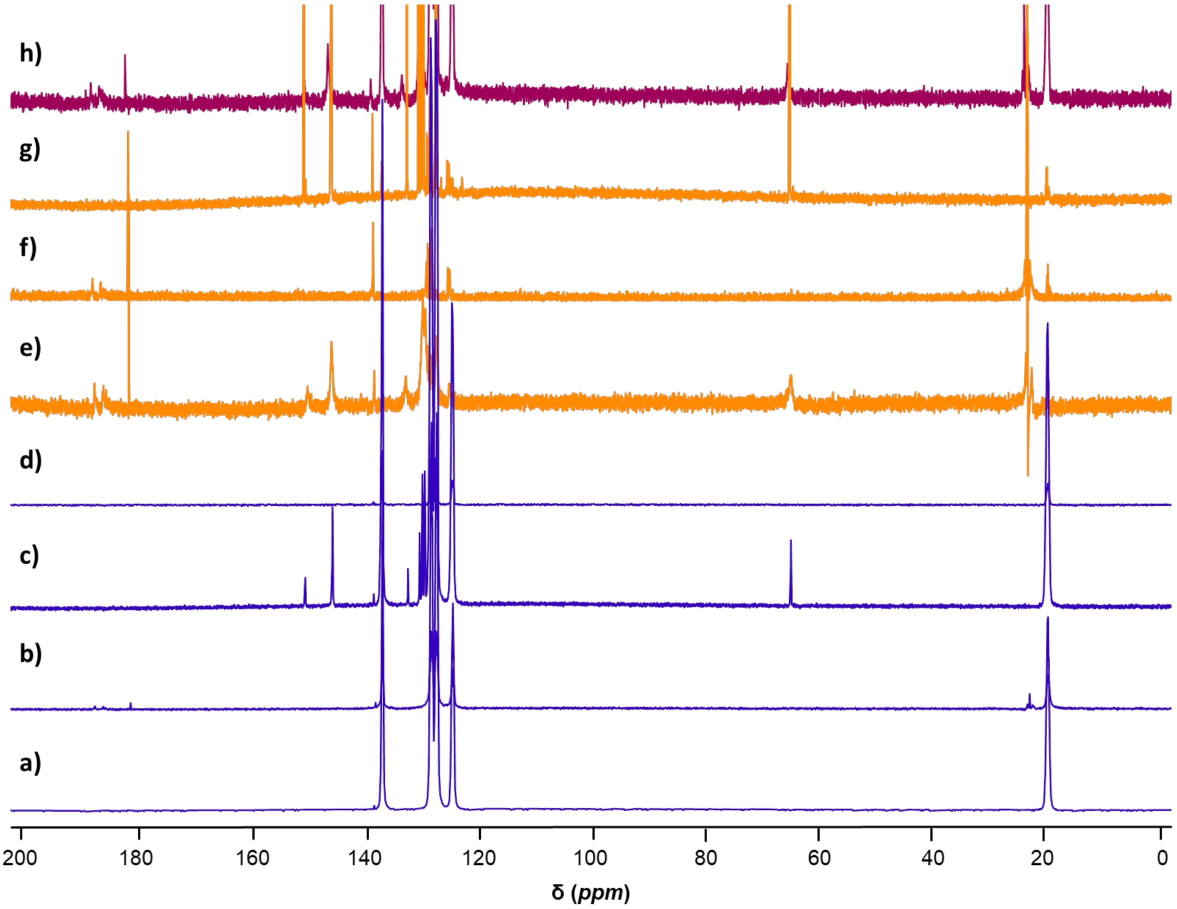

Figure S20: ^{13}C NMR spectrum of **a)** 500 µL D_2O and 200 µL toluene-d_8; **b)** 1 eq. $\{Pd_{84}\}^{Ac}$ in 500 µL D_2O and 200 µL toluene-d_8; **c)** 7 eq. BV^{2+} in 500 µL D_2O and 200 µL toluene-d_8; **d)** 1 eq. C_{60}-fullerene in 500 µL D_2O and 200 µL toluene-d_8; **e)** $\{Pd_{84}\}^{Ac}$ and 7 eq. BV^{2+} in 500 µL D_2O and 200 µL toluene-d_8; **f)**

$\{Pd_{84}\}^{Ac}$ and 1 eq. C_{60}-fullerene in 500 µL D_2O and 200 µL toluene-d_8; **g)** BV^{2+} and 1 eq. C_{60} in 500 µL D_2O and 200 µL toluene-d_8 and **h)** $\{Pd_{84}\}^{Ac}$ and 7 eq. BV^{2+} and 1 eq. C_{60}-fullerene in 500 µL D_2O and 200 µL toluene-d_8. (600 MHz, D_2O/toluene-d_8, 298 K).

4.5 Experimental Procedures for ^{13}C NMR Work on 3-Component System

The limiting factor in experiments using $\{Pd_{84}\}^{Ac}$, BV^{2+} and C_{60}-fullerene is the mass of C_{60}-fullerene that can be dissolved in 200 µL toluene-d_8.

Solubility of C_{60}-fullerene in toluene-d_8 = 3 gL^{-1} = 3 mg mL^{-1} = *0.003 mg µL^{-1}*

Therefore it is possible for 0.6 mg of C_{60}-fullerene to be dissolved in 200 µL of solvent.

1 equivalent C_{60}-fullerene = 0.83 x 10^{-3} mmol = **0.6 mg**

1 equivalent $\{Pd_{84}\}^{Ac}$ = 0.83 x 10^{-3} mmol = **16.8 mg**

7 equivalent BV^{2+} = 0.83 x 10^{-3} mmol = **2.38 mg**

4.6 ¹H-¹H NOESY NMR Data

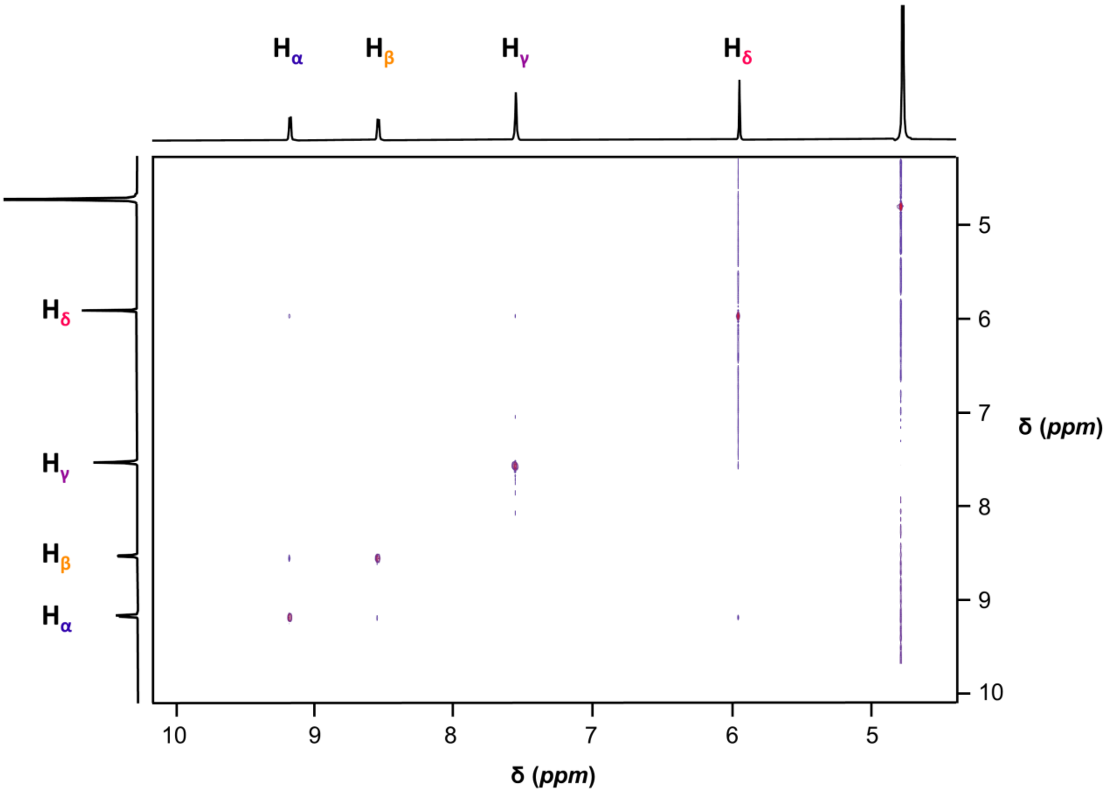

Figure S21: ¹H-¹H NOESY of BV^{2+} (600 MHz, D_2O, 298 K).

4.7 Molecular Visualisation of 3-Component System

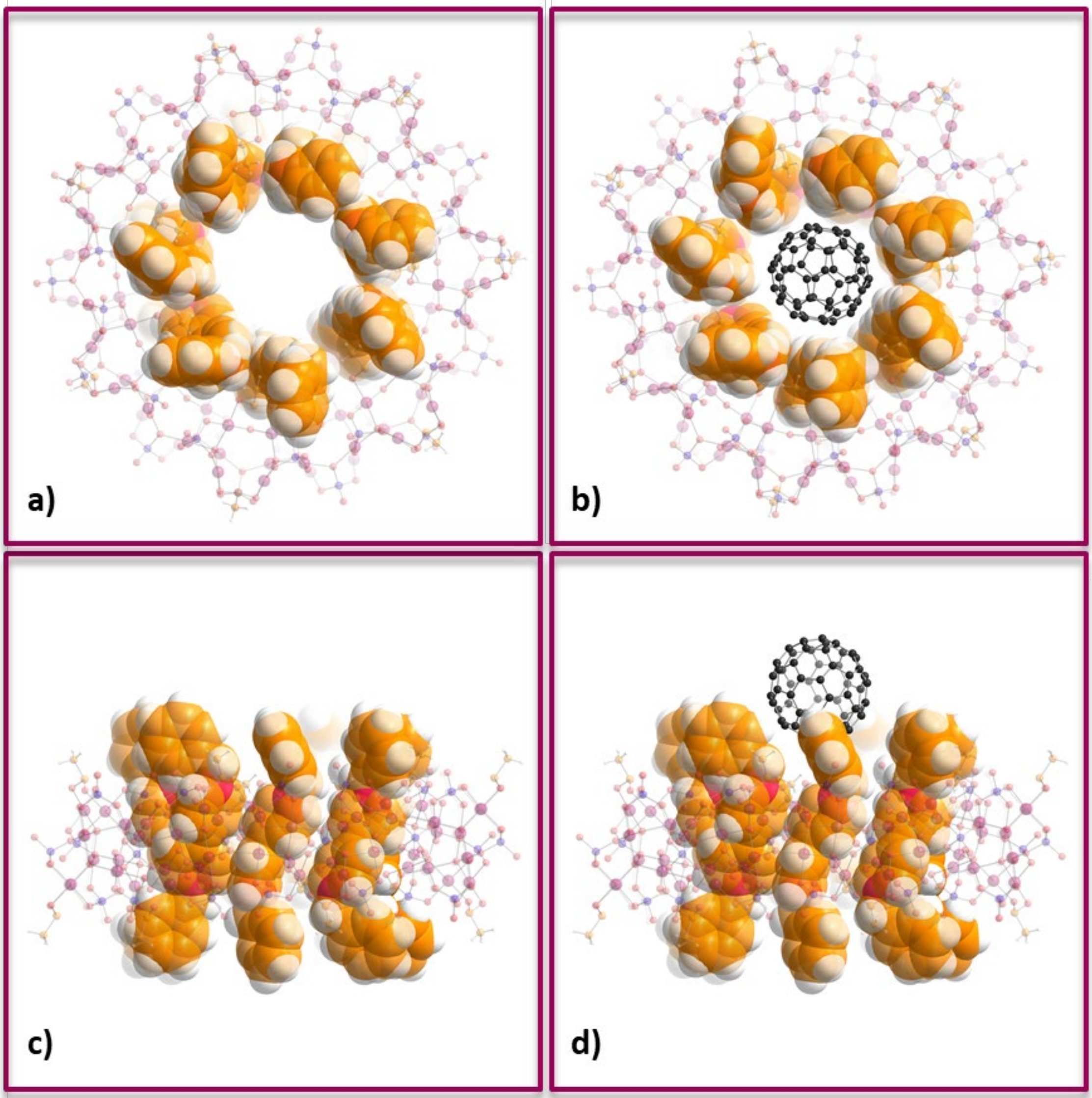

Figure S22: Molecular visualisation models of a) $\{Pd_{84}\}^{Ac}$ host with 7 BV^{2+} guests from face-on, b) $\{Pd_{84}\}^{Ac}$ host with 7 BV^{2+} guests and C_{60}-fullerene from face-on, c) $\{Pd_{84}\}^{Ac}$ host with 7 BV^{2+} guests from a side-on perspective and d) $\{Pd_{84}\}^{Ac}$ host with 7 BV^{2+} guests and C_{60}-fullerene from a side-on perspective.

References

[1] F. Xu, H. N. Miras, R. A. Scullion, D.-L. Long, J. Thiel, L. Cronin, *Proc. Natl. Acad. Sci.* **2012**, *109*, 11609–11612.

[2] L. G. Christie, A. J. Surman, R. A. Scullion, F. Xu, D. L. Long, L. Cronin, *Angew. Chemie - Int. Ed.* **2016**, *55*, 12741–12745.

[3] R. A. Scullion, A. J. Surman, F. Xu, J. S. Mathieson, D. L. Long, F. Haso, T. Liu, L. Cronin, *Angew. Chemie - Int. Ed.* **2014**, *53*, 10032–10037.

[4] R. B. Bedford, J. G. Bowen, R. B. Davidson, M. F. Haddow, A. E. Seymour-Julen, H. A. Sparkes, R. L. Webster, *Angew. Chemie Int. Ed.* **2015**, *54*, 6591–6594.

[5] L. Fielding, *Tetrahedron* **2000**, *56*, 6151–6170.

[6] K. S. Cameron, L. Fielding, *J. Org. Chem.* **2001**, *66*, 6891–6895.
